# Supershed *Escherichia coli* O157:H7 Has Potential for Increased Persistence on the Rectoanal Junction Squamous Epithelial Cells and Antibiotic Resistance

**DOI:** 10.1155/2020/2368154

**Published:** 2020-04-13

**Authors:** Raies A. Mir, Brian W. Brunelle, David P. Alt, Terrance M. Arthur, Indira T Kudva

**Affiliations:** ^1^Food Safety Enteric Pathogens Research Unit (FSEPRU), National Animal Disease Center (NADC), ARS/USDA, Ames, IA 50010, USA; ^2^Oak Ridge Institute for Science and Education (ORISE), Oak Ridge, TN 37831, USA; ^3^Infectious Bacterial Disease Research Unit (Genomics Center), NADC, ARS/USDA, Ames, IA 50010, USA; ^4^U.S. Meat Animal Research Center, ARS/USDA, Spur 18D, Clay Center, NE 68933, USA

## Abstract

Supershedding cattle shed *Escherichia coli* O157:H7 (O157) at ≥ 10^4^ colony-forming units/g feces. We recently demonstrated that a supershed O157 (SS-O157) strain, SS-17, hyperadheres to the rectoanal junction (RAJ) squamous epithelial (RSE) cells which may contribute to SS-O157 persistence at this site in greater numbers, thereby increasing the fecal O157 load characterizing the supershedding phenomenon. In order to verify if this would be the signature adherence profile of any SS-O157, we tested additional SS-O157 isolates (*n* = 101; each from a different animal) in the RSE cell adherence assay. Similar to SS-17, all 101 SS-O157 exhibited aggregative adherence on RSE cells, with 56% attaching strongly (>10 bacteria/cell; hyperadherent) and 44% attaching moderately (1–10 bacteria/cells). Strain typing using Polymorphic Amplified Typing Sequences (PATS) analysis assigned the 101 SS-O157 into 5 major clades but not to any predominant genotype. Interestingly, 69% of SS-O157 isolates were identical to human O157 outbreak strains based on pulsed field gel electrophoresis profiles (CDC PulseNet Database), grouped into two clades by PATS distinguishing them from remaining SS-O157, and were hyperadherent on RSE cells. A subset of SS-O157 isolates (*n* = 53) representing different PATS and RSE cell adherence profiles were analyzed for antibiotic resistance (AR). Several SS-O157 (30/53) showed resistance to sulfisoxazole, and one isolate was resistant to both sulfisoxazole and tetracycline. Minimum inhibitory concentration (MIC) tests confirmed some of the resistance observed using the Kirby–Bauer disk diffusion test. Each SS-O157 isolate carried at least 10 genes associated with AR. However, genes directly associated with AR were rarely amplified: *aac (3)-IV* in 2 isolates, *sul*2 in 3 isolates, and *tetB* in one isolate. The integrase gene, *int*, linked with integron-based AR acquisition/transmission, was identified in 92% of SS-O157 isolates. Our results indicate that SS-O157 isolates could potentially persist longer at the bovine RAJ but exhibit limited resistance towards clinical antibiotics.

## 1. Introduction

*Escherichia coli* O157:H7 (O157) was the first Shiga toxin-producing *Escherichia coli* (STEC) serotype to be associated with bloody diarrhea or hemorrhagic colitis (HC) and hemolytic uremic syndrome (HUS) in humans [1, 2]. It was isolated 36 years ago, in 1982, from contaminated hamburgers that caused a two-state outbreak in the United States (US) [3] and has since been annually implicated in an estimated 63,153 illnesses, 2,138 hospitalizations, and 20 deaths in humans, in the US alone [4–6].

Cattle are the primary reservoirs and asymptotic carriers of O157, which preferentially colonize at the rectoanal junction (RAJ) [5]. In the US, O157 prevalence ranges from 0.2 to 48.8% in dairy and 0.2 to 27.8% in beef cattle [7–11]. Cattle shed O157 seasonally, with increased shedding in warmer months and decreased shedding in winter [12]. Some animals intermittently shed greater than 10^4^ CFU/g feces of O157 and are termed “supershedders” [12–14] with the corresponding O157 strains referred to as supershed O157 (SS-O157) [13]. STEC survival on farms is well documented [13, 14], and supershedder cattle increase the number of O157 in pens and thereby enhance herd prevalence on farms and feedlots [15]. Supershedding phenomenon may be influenced by host, bacterial, and/or environmental factors [12].

Few studies conducted thus far have been in the context of bacterial factors as it relates to supershedding. One study associated phage-type PT 21/28, linked with increased morbidity in humans, with SS-O157 strains [15–17]. Arthur et al. found 71% of a genetically diverse set of 102 SS-O157 strains to have a substitution of an A nucleotide for a T at position 255 of the translocated Intimin receptor or *tir* gene, a mutation that was identified in human clinical isolates [18]. We recently demonstrated that SS-O157 strain SS-17, one of the 102 SS-O157 isolates, hyperadheres (aggregative, strong adherence pattern) to the bovine rectoanal junction (RAJ) squamous epithelial (RSE) cells using mechanisms independent of the adhesin Intimin, which may contribute to SS-O157 persistence at this site in greater numbers [18, 19]. Sequence analysis of SS-O157 strain SS-17 identified several nonsynonymous single nucleotide polymorphisms (SNPs) in virulence and adherence genes such as those encoding nonfimbrial adhesins *cah, yfaL*, and *toxB* [18, 19] that may contribute to the increased adherence observed with this strain. Comparative analyses of the SS-17 genome with that of another hyperadherent SS-O157 strain SS-52 revealed 167 nonsynonymous SNPs in different virulence and adherence genes that will require further analyses to ascertain their role in supershedding [20].

Antibiotic treatment of STEC infections in humans is currently not advocated in the US, with some studies suggesting that treatment may exacerbate toxin-related tissue damage and symptoms in patients [21]. However, a recent study found that overall it is not the antibiotic treatment, but rather the type of antibiotic (e.g., *β*-lactam antibiotics) used within 3 days of diarrhea that could be associated with the development of HUS [22]. Antibiotics such as rifaximin, fosfomycin, azithromycin, and meropenem were found to not stimulate the release of Shiga toxin from O157 and non-O157 strains in vitro [23, 24]. These antibiotics have been recommended for the treatment of early stages of STEC disease to prevent HUS [25–27]. However, resistance to antibiotics in STEC could confound pursuing these options. Antibiotic resistance (AR) in STEC isolates varies considerably depending on the host species (animal versus human) and source of isolation [28, 29], but incidences as low as 6% (from cattle feces) [30] to as high as 58% (from dairy products) [31] have been reported. Also, multidrug resistant STEC has been isolated from calf feces [32], beef, and dairy products [31]. Plasmids carrying integrons, which are gene-capture systems, play an important role in acquisition/transmission of antimicrobial resistance genes and have been found in several O157 as well as non-O157 STEC [32]. Considering that SS-O157 persists in the bovine host, they are exposed to the same selection pressures as other bacteria in the intestine allow for increased acquisition/transmission of AR. While antibiotics are not used to clear STEC in cattle, there could be collateral effects with transmission of AR genes from STEC to other bacteria and since SS-O157 isolates increase O157 load in the environment [13, 15–17], they could also contribute towards increased dissemination of antibiotic resistance.

Phenotypic characterization of AR in bacterial isolates can be determined both qualitatively, using the antibiotic sensitivity test (AST), and quantitatively, using the minimum inhibitory concentration (MIC) test, both of which are based on the Kirby–Bauer disk diffusion method [33]. It is important to know prevalence (by AST) as well as level of resistance (by MIC) to an antibiotic to achieve successful treatment of bacterial infection; the Clinical Laboratory Standards Institute (CLSI) updates and modifies AST and MIC guidelines through a global consensus process to ensure uniformity of technique and reproducibility of results across different laboratories, making these tests universally applicable tools to determine antibiotic resistance [34, 35]. Genotypic characterization of most AR in bacteria relies on the demonstration of the presence of AR genes on the chromosome or plasmid by polymerase chain reaction (PCR), and various modifications of PCR have been successfully employed to detect AR genes in bacteria obtained from humans, animals, or environment [36–38]. The presence of a resistance gene in an isolate may affect the clinical outcome of antimicrobial therapy. For instance, European countries have used macrolides like azithromycin in STEC treatment in early stages of human infection without any induction of Shiga toxin expression but the *mph(A)* gene that confers resistance to azithromycin could prevent successful application of this therapy [39]. Most common AR genes observed in STEC are isolated from humans, *blaTEM-1, strA, strB, sul1, sul2, dfrA, and tet(A)* [40], while *floR, ampC, tet(A), blaTEM,* and *sul1* have been identified in bovine STEC isolates recovered from farms and abattoirs [41]. In addition, resistance to antibiotics like ampicillin, gentamicin, streptomycin, sulfisoxazole, tetracycline, and trimethoprim-sulfamethoxazole has been associated with the presence of class 1 integrons [42].

In this study, we sought to verify (i) if aggregative adherence would be the signature adherence profile of any SS-O157, (ii) if SS-O157 demonstrate AR and carry AR genes, and (iii) if SS-O157 strains demonstrate genetic relatedness and profiles that can be linked to either the adherence and/or AR phenotypes. To determine the adherence phenotype, previously characterized [18] SS-O157 isolates ((*n* = 101), each from a different animal) were tested in the RSE cell adherence assay. In addition, we used phenotypic (AST and MIC) and genotypic tests to characterize AR in the SS-O157 isolates, in accordance with the CLSI guidelines [34], against antibiotics important in human clinics and previously reported AR genes in STEC isolates from humans and from animals, farms, and food sources [40, 41]. The presence of tetracycline resistance genes in SS-O157 isolates was also investigated based on their frequent prevalence in O157 isolates from humans and cattle [29]. The integrase gene, *int*, linked with integron-based AR acquisition/transmission, and the colistin resistance encoding genes *(mcr1*, *mcr2)* have attracted much interest from the scientific community [42–44], and hence, we aimed to determine the presence of these genes in our set of SS-O157 isolates. These data are useful because the presence of a resistance gene in pathogens could affect the clinical outcome of antimicrobial therapy and enable the spread of AR in the environment or within reservoir hosts. Considering the inherent genetic variations between O157 strains [45–47], we used the rapid, PCR-based, Polymorphic Amplified Typing Sequences [48–51] typing system to group genetically related SS-O157 isolates and sought to correlate it to any of the phenotypes being characterized.

## 2. Materials and Methods

### 2.1. Bacterial Strains

Previously isolated SS-O157 (*n* = 101) were used in this study [18]. Control O157 strains included SS-O157 and O157 that have been sequenced, namely: SS-17 [19, 20], SS-52 [18], JEONG-1266 (provided by Dr. K. C. Jeong, University of Florida, Gainesville FL), EDL 933 (ATCC®43895™) and Sakai (ATCC®BAA-460™) obtained from the American Type Culture Collection (ATCC; Manassas, VA), and EC4115 (STEC Center, Michigan State University, East Lansing, MI). A non-STEC *E. coli* (ATCC®25922™) was included as control only in antibiotic susceptibility tests per CLSI recommendations [34].

Twenty generic bovine *E. coli* (non-O157) were isolated from fecal samples collected from five healthy, nonchallenged, control cows enrolled in another study (NADC Institutional Animal Care and Use Committee protocol # ARS-2016-480). Fecal samples (1 g) were inoculated in 10 mL of trypticase soy broth (Becton Dickinson, Franklin Lakes, NJ) and incubated at 37°C for 18 h. The overnight fecal cultures were plated (100 *μ*L) on MacConkey agar plates, and four random pink colonies were selected per sample, each from a different plate, and confirmed as *E. coli* by Gram staining, growth on CHROMagar™ *E. coli* (DRG International Inc., Springfield, NJ), and biochemical tests using analytical profile index API 20E test strips (BioMe'rieux Inc., Durham, NC). Four *E. coli* isolates per fecal sample were selected to cover any genetic variability among *E. coli* isolates from the same animal. All 20 bovine *E. coli* isolates were tested for O157 and six non-O157 STEC (O26, O45, O103, O111, O121, and O145) antigens using the latex agglutination tests (Oxoid/Thermo Scientific Pierce, Logan, UT).

Select SS-O157 isolates (*n* = 53) representing the different PATS and RSE cell adherence profiles were used in AST assays and AR gene PCR, as described below, to determine their phenotypic and genetic antibiotic resistance profile, as described below. All isolates that were either resistant (R) or intermediate resistant (I) in AST assays were confirmed by MIC testing. Six control O157 strains and 20 bovine *E. coli* were also used in these assays. PCR followed by sequencing was done to determine the presence of AR genes.

### 2.2. Polymorphic Amplified Typing Sequences- (PATS-) Based Typing and Categorization of SS-O157

Each colony lysate was tested in triplicate to confirm the profiles generated as described previously [49–51]. Briefly, primer pairs targeting the 8 polymorphic *Xba*I-, 7 polymorphic *Avr*II-restriction enzyme sites, and the four virulence genes encoding the Shiga toxin 1 and 2 (*Stx*1 and *Stx*2), Intimin-*γ* (*eae*), and hemolysin-A (*hly*A) were used to generate amplicons from the colony lysates in a hot start, touchdown PCR reaction [49–51]. PCR reactions amplifying the *Avr*II- restriction enzyme site were purified using the QIAquick PCR purification kit (Qiagen, Valencia, CA) and digested with the *Avr*II-restriction enzyme (New England Biolabs, Beverly, MA) to confirm the presence of the restriction site.

The unweighted pair group method with arithmetic means (UPGMA) algorithm was used to create dendrograms based on the PATS profile of all SS-O157 (*n* = 101) using the Molecular Evolutionary Genetics Analysis software version 7 (MEGA 7.0; http://www.megasoftware.net/). The presence or absence of each amplicon was used in coding and creating dendrograms as follows: absence of an amplicon (score, 0) to the presence of an amplicon (score, 1) and the additional presence of a single functional *AvrII* site (score, 2). The same scoring pattern was used to represent the PATS data graphically as Minimum Spanning Trees using the PHYLOViZ software (http://www/phyloviz.net/), described as follows.

### 2.3. Adherence Assays

For adherence assays, bacterial strains were cultured overnight in Dulbecco's modified Eagle's medium with low glucose, DMEM-LG (Invitrogen, Carlsbad, CA) at 37°C without aeration, washed, and resuspended in DMEM with no glucose (DMEM-NG; Invitrogen) as described previously [19, 52]. To determine the adherence patterns of bacterial isolates on eukaryotic cells, two adherence assays using two different types of cells were carried out, following standardized protocols [19, 52–56]:

#### 2.3.1. Rectoanal Junction Squamous Epithelial (RSE) Cell Assay

The RSE assay was done with 4 technical and 2 biological replicates. As previously described [52–56], RSE cells were collected from the rectoanal junctions of cattle included in unrelated experiments at the National Animal Disease Center (NADC, Ames, IA.), under the approval of the NADC Institutional Animal Care and Use Committee and stored at −80°C. RSE cells were suspended in DMEM-NG to a final concentration of 10^5^ cells/ml. Each bacterial isolate was mixed with RSE cells at a bacteria°:°cell ratio of 10 : 1. The mixture was incubated at 37°C with aeration (110 rpm) for 4 h, pelleted, washed, and reconstituted in 100 *μ*l of double-distilled water (dH_2_O). Drops of the suspension (2 *μ*l) were placed on Polysine slides (Thermo Scientific/Pierce, Rockford, IL), dried, fixed, and stained with fluorescence-tagged antibodies specific to the O157 antigen and cytokeratins of the RSE cells as previously described [52–56]. Adherence patterns on RSE cells were qualitatively recorded as diffuse, aggregative, or nonadherent and quantitatively as the percentages of RSE cells with or without adhering bacteria [55]; adherence was recorded as strongly adherent (hyperadherent) when more than 50% of RSE cells had 10 adherent bacteria, moderately adherent when 50% or less of the RSE cells had 5 to 10 adherent bacteria, and nonadherent when less than 50% of the RSE cells had only 1 to 5 adherent bacteria. RSE cells with no added bacteria were subjected to the assay procedure and used as negative controls to confirm the absence of preexisting O157 bacteria.

#### 2.3.2. HEp-2 Cell Adherence Assay

Adherence patterns displayed by bacterial strains on HEp-2 cells (human epidermoid carcinoma of the larynx cells with HeLa contamination) (ATCC® CCL-23™) were determined using the same growth conditions as those used for the RSE adherence assay and as described previously [19, 52–56]. The Hep-2 adherence assay was performed with two technical and 2 biological replicates per bacterial strain. Besides the control strains, only 53/101 SS-O157 isolates representing all major clades in PATS and RSE cell adherence profiles were tested in this assay. Slides were stained with fluorescence-tagged antibodies that target the O157 antigen and the HEp-2 cell actin filaments as described previously [52, 55] and qualitatively and quantitatively recorded as indicated above.

### 2.4. Antibiotic Susceptibility Testing (AST)

Fifty-three SS-O157 isolates were selected based on their PATS and RSE cell adherence profiles, representing all the clades for maximum genetic diversity and varying adherence phenotypes. For comparison, the six control O157 (SS17, SS52, JEONG-1266, EDL933, Sakai, and EC4115) strains and 20 bovine *E. coli* isolates were also included. All isolates were tested for resistance to 17 clinically relevant antibiotics in a Kirby–Bauer disk diffusion method according to the Clinical and Laboratory Standards Institute (CLSI) guidelines [34] ([Supplementary-material supplementary-material-1]). In short, purified bacterial colonies [3–5] were inoculated in trypticase soy broth (TSB; Becton Dickinson/Thermo Fisher Scientific, Rockford, IL) to reach a McFarland turbidity of ∼0.5 (concentration ∼10^5^ CFU/mL). The inoculum was spread-plated onto Mueller-Hinton Agar (MHA) (Becton Dickinson/Thermo Fisher Scientific) plates using a sterile cotton swab (Thermo Fisher Scientific). Within 15 min of inoculation, the antibiotic discs were dispensed onto MHA plates using the automatic disc dispenser (Becton Dickinson/Thermo Fisher Scientific). The plates were incubated at 37°C for 16–18 h and the isolates were classified as susceptible (S), intermediate resistant (I), or resistant (R) based on the diameter of the zone of inhibition around each disc ([Supplementary-material supplementary-material-1]) and the interpretive criteria from the CLSI manual [34]. The following antibiotic discs were used: Ampicillin (10 *μ*g), Amoxicillin/Clavulanic acid (20/10 *μ*g), Azithromycin (15 *μ*g), Cefoxitin (30 *μ*g), Ceftiofur (30 *μ*g), Ceftriaxone (30 *μ*g), Ciprofloxacin (5 *μ*g), Chloramphenicol (30 *μ*g), Colistin (10 *μ*g), Fosfomycin (200 *μ*g), Gentamicin (10 *μ*g), Nalidixic acid (30 *μ*g), Polymyxin B (300 IU), Streptomycin (10 *μ*g), Sulfisoxazole (0.25 mg), Sulfamethoxazole/Trimethoprim (23.75/1.25 *μ*g), and Tetracycline (30 *μ*g) (Becton Dickinson/Thermo Fisher Scientific). The CLSI recommended reference strain for antibiotic susceptibility testing, *E. coli* (ATCC®25922™), was used to validate assay conditions and quality of antibiotics [34].

AST profile of the SS-O157 isolates was represented graphically as Minimum Spanning Trees with an N locus variant of 0, either by itself or in combination with the PATS profiles, using the PHYLOViZ 2.0 software (http://www/phyloviz.net/) and by converting the results obtained with each antibiotic into scores as follows: resistant (score, 0), intermediate resistant (score, 1), and susceptible (score, 2).

### 2.5. Minimum Inhibitory Concentration (MIC) Testing

All bacterial isolates (53 SS-O157, 6 control O157 and 20 bovine *E. coli*) with intermediate-resistant and resistant AST phenotype were selected for MIC testing against respective antibiotics using the MIC strips (Etest®, BioMe'rieux Inc., Durham NC). Hence, isolates were tested for MIC against 11 antibiotics, in triplicate for each antibiotic (results represented as average MIC *μ*g/mL), based on the CLSI guidelines [34] ([Supplementary-material supplementary-material-1]). Briefly, four purified bacterial colonies were inoculated in trypticase soy broth (TSB; Becton Dickinson/Thermo Fisher Scientific, Rockford, IL) to reach a McFarland turbidity of ∼0.5 (concentration, ∼10^5^ CFU/mL). The inoculum was spread-plated onto the Mueller-Hinton Agar (MHA) (Becton Dickinson/Thermo Fisher Scientific) plates using sterile cotton swabs (Thermo Fisher Scientific). Within 15 min of inoculation, the MIC Strips (Etest®) were placed onto MHA plates. The plates were incubated at 37°C for 16–18 h and the MIC (*μ*g/mL), corresponding to the elliptical zone of inhibition around the strip ([Supplementary-material supplementary-material-1]), was directly read from the strips. *E. coli* (ATCC®25922™) was used as a control to validate assay conditions and the quality of antibiotics [34].

### 2.6. PCR Screening and Sequence Analysis of Antibiotic Resistance, Integrase, and Shiga Toxin Genes

#### 2.6.1. PCR Screening

All bacterial isolates (53 SS-O157, 6 control O157 and 20 bovine *E. coli*) were screened by PCR for 31genes directly or indirectly associated with AR, the integrase (*int*), the colistin resistance (*mcr-1, mcr-2*), and the Shiga toxin-1 (*stx*1) and -2 (*stx*2) genes. Colony lysates prepared from isolated bacterial colonies on trypticase soy agar (TSA; Difco, Becton Dickenson, Sparks, MD) were used as a template. Target-specific primers were generated using the Primer-BLAST software [57] or derived from other studies as shown in [Supplementary-material supplementary-material-1]. Each colony lysate was tested with individual primer pairs. PCR was carried out on the ABI GeneAmp 9700 PCR thermal cycler (Applied Biosystems, Foster City, CA) using 10 *μ*l of colony lysate, 200 pmol of each primer, 800 *μ*M deoxynucleoside triphosphates, 1X diluted Ex *Taq* enzyme buffer, and 2.5 U of TaKaRa Ex *Taq* DNA polymerase (Takara Bio Inc., Mountain View, CA). The hot-start PCR technique was employed in combination with touchdown PCR [58] spanning the annealing temperature range of 60°C to 40°C for the initial 20 cycles. Then, another amplification segment of 15 cycles was set using 40°C as the final annealing temperature. The amplified products were resolved on 1% agarose gel stained with ethidium bromide and visualized with a UV gel doc system (Bio-Rad, Hercules, CA).

#### 2.6.2. Sequencing

PCR amplicons were gel extracted and purified using the QIAquick Gel Extraction Kit (Qiagen, USA) and sequenced using the Applied Biosystems BigDye v3.1 Terminator chemistry on an ABI 3130xl instrument at the Infectious Bacterial Disease Research Unit (Genomics Center), NADC, Ames, IA. Each PCR product was sequenced using forward and reverse PCR primers ([Supplementary-material supplementary-material-1]). Nucleotide sequences were analyzed using EditSeq™ version 14.0.0 and MegAlign Pro™ version 14.0.0 (DNASTAR® Madison, WI) and compared with sequences available at https://blast.ncbi.nlm.nih.gov/Blast.cgi [59, 60]. A consensus sequence was generated using the forward sequence and reverse complement of the reverse sequence before analyzing for homology/identity with previously reported genes in the NCBI database using the Nucleotide Basic Local Alignment Search Tool (BLASTn) [60]. PCR products/amplicons corresponding to *mcr-1* primers (short- and full-length) observed after PCR from 11 bacterial isolates were also analyzed by sequencing.

The *Stx2* gene was identified in four bovine *E. coli* isolates from one animal (animal #887) amplified *Stx2* gene and these sequences were compared with *Stx2* sequences from control O157 strains. In total, we sequenced 10 *Stx2* amplicons and the gene sequences aligned using Clustal W prior to the phylogenetic analysis conducted using MEGA7 [59]. Phylogenetic analysis (neighbor joining tree) of *stx*2 sequences, the genetic distance (evolutionary divergence) and rate variation among sites analyses were conducted using the maximum likelihood statistical method and Jukes-Cantor nucleotide substitution model with a gamma distribution (JC + G model, shape parameter = 0.5). Group names (SS O157, control O157, and *E. coli*) were added to the sequences to designate the origin. The maximum likelihood phylogenetic tree was constructed from the sequences using the JC + G model and 1000 bootstrap replications.

## 3. Results

### 3.1. Polymorphic Amplified Typing Sequences (PATS) Categorized 101 SS-O157 Isolates into Five Distinct Clades

**Figure 1:**
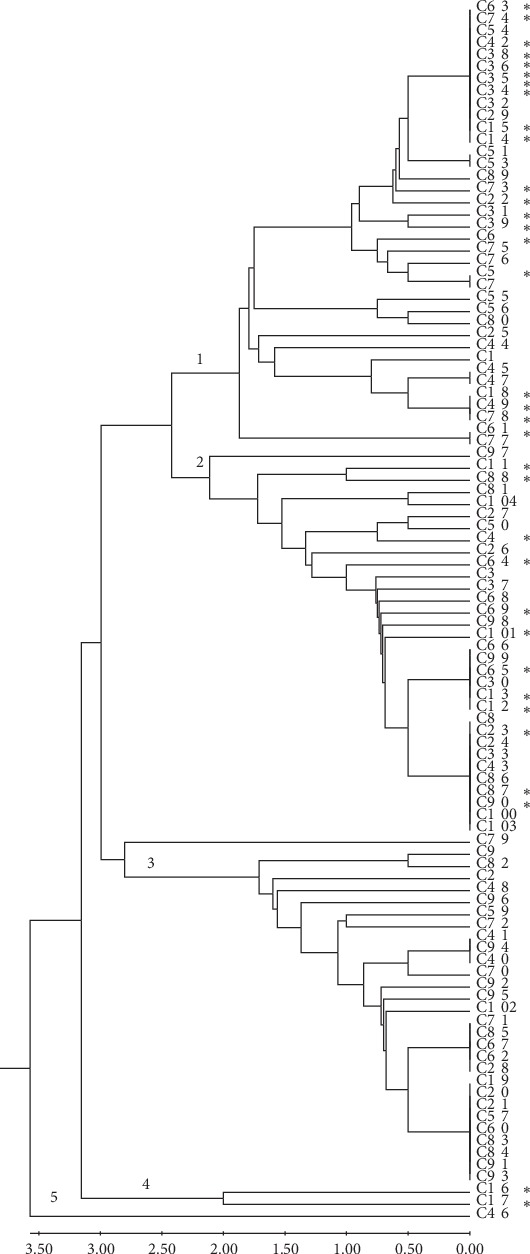
Dendrogram based on the PATS profiles obtained for the SS-O157 (*n* = 101) and constructed using the unweighted pair group method with arithmetic means (UPGMA) algorithm using the Molecular Evolutionary Genetics Analysis software version 7 (MEGA 7.0; http://www.megasoftware.net/). The numbers in red indicate the five major clades.

PATS analysis verified the genetic diversity of the SS-O157 isolates tested (*n* = 101, [Supplementary-material supplementary-material-1]). The dendogram generated with the MEGA 7.0 software, based on the PATS profiles, categorized SS-O157 isolates into five clades of which Clade 1, 2 and 3 comprised 35, 33 and 30 isolates, respectively (Figure 1, [Supplementary-material supplementary-material-1]). Arthur et al. in their study found 36 of the 101 SS-O157 to have identical pulsed field gel electrophoresis (PFGE) patterns as O157 associated with human outbreaks by the Centers for Disease Control and Prevention (CDC) and stored in Pulse Net data base [18, 61, 62]. In our analysis, SS-O157 having identical PFGE patterns as that of human outbreak strains (34/36, 94%) were restricted mainly to Clades 1 and 2 (Figure 1) reflecting their possible distinction from other SS-O157 isolates even though all SS-O157 isolates have an animal/bovine origin and are clonal in nature [48, 63].

### 3.2. All SS-O157 Demonstrated an Aggregative Adherence Phenotype on RSE Cells Compared to HEp-2 Cells

**Figure 2:**
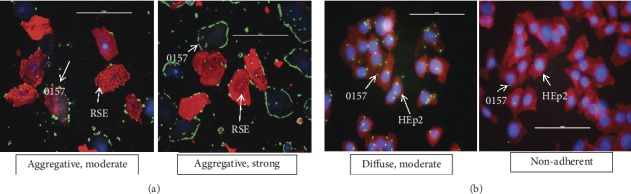
Representative adherence patterns of SS-O157 strains on RSE cells (a) and HEp-2 cells (b). (a) The “aggregative, moderate” and “aggregative, strong” adherence patterns seen with RSE cells is shown. (b) The “diffuse, moderate” and “nonadherent” adherence patterns seen with HEp-2 cells are shown. The immunofluorescence stained slides are shown at 40x magnification. Bacteria (O157) have green fluorescence, RSE cells' cytokeratins and HEp-2 cells' actin filaments have orange-red fluorescence, and the nuclei of both cells have blue fluorescence.

To determine if a common adherence phenotype contributes to the high numbers of SS-O157 shed by cattle isolates were evaluated in RSE and HEp-2 cell adherence assays [52–56]. Of the SS-O157 tested (*n* = 101), 54% demonstrated an aggregative-strong or hyperadherent (>50% RSE cells have >10 bacteria/cell) adherence phenotype and 44% were aggregative-moderate (>50% RSE cells have 5–10 bacteria/cell) on RSE cells ([Supplementary-material supplementary-material-1], Figure 2). Although not all SS-O157 isolates were hyperadherent they collectively shared the aggregative adherence phenotype on RSE cells ([Supplementary-material supplementary-material-1], Figure 2). However, the hyperadherent trait could contribute to the increased persistence of SS-O157 in cattle. Interestingly, of the 36 SS-O157 that were identical to human outbreak strains by PFGE profiles (CDC PulseNet Database; 18), 69% (25/36) were hyperadherent, while 31% (11/36) were aggregative-moderate in their adherence to the RSE cells. As reported previously, the results of HEp-2 cell adherence assay did not provide any insights into the differential adherence capabilities of SS-O157, confirming that these nongastrointestinal cells do not reflect the true bacterial-host interactions that likely occur in the gastrointestinal tract (GIT) of either humans or cattle [19, 52–56]. Of the SS-O157 isolates (*n* = 53) tested, 89% (47/53) demonstrated a diffuse-moderate adherence phenotype as seen with most O157 isolates [19, 49–53] and 11% (6/53) were nonadherent on HEp-2 cells ([Supplementary-material supplementary-material-1], Figure 2).

Control strains showed similar host specific adherence profiles. Five of the six control O157 (SS17, SS52, JEONG-1266, EDL933, and EC4115) strains demonstrated aggregative adherence on RSE cells with the exception of one strain (Sakai) which adhered in a diffuse pattern ([Supplementary-material supplementary-material-1]). However, all six strains adhered to HEp2 cells in a diffuse manner ([Supplementary-material supplementary-material-1]).

### 3.3. SS-O157 Demonstrated Varying Susceptibility to the 17 Antibiotics Evaluated Using Antibiotic Susceptibility Tests (AST)

Of the 53 SS-O157 isolates tested in the AST assay, 57% (30/53) were resistant to sulfisoxazole (0.25 *μ*g), and one isolate, C104, was resistant to both sulfisoxazole (0.25 *μ*g) and tetracycline (30 *μ*g) ([Supplementary-material supplementary-material-1]). Also, 89% (47/53), 69% (37/53), 26% (14/53), 22% (12/53), and 9% (5/53) of the isolates had intermediate resistance to azithromycin (AZM-15), streptomycin (S-10), chloramphenicol (C-30), ceftiofur (XNL-30), and tetracycline (TE-30), respectively ([Supplementary-material supplementary-material-1]). Antibiograms of the 6 control O157 strains indicated resistance in 50% (3/6) and intermediate resistance in 50% (3/6) of the strains to azithromycin ([Supplementary-material supplementary-material-1]). Also, intermediate resistance was observed in 50, 83, 66, 50, and 50% control strains against sulfisoxazole (G-25), chloramphenicol (C-30), tetracycline (TE-30), streptomycin (S-10), and cefoxitin (FOX-30), respectively ([Supplementary-material supplementary-material-1]).

The bovine *E. coli* isolates (*n* = 20) also demonstrated varying susceptibility to the 17 antibiotics tested with 40, 30, 15, and 15% of the *E. coli* being resistant to sulfisoxazole, tetracycline, chloramphenicol, and streptomycin, respectively ([Supplementary-material supplementary-material-1]). The antibiogram of bovine *E. coli* strains indicated 5% of isolates to be resistant to gentamicin and azithromycin. Intermediate resistance was observed in 75, 40, 25, and 25% of the isolates against the streptomycin, tetracycline, amoxicillin/clavulanic acid, and ampicillin, respectively (Tables [Supplementary-material supplementary-material-1] and [Supplementary-material supplementary-material-1]).

**Figure 3:**
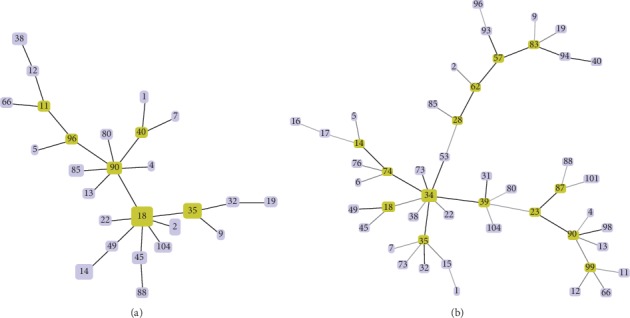
Minimum spanning trees constructed using the PHYLOViZ 2.0 software (http://www/phyloviz.net/), with an N locus variant of 0, and the AST data (a) or the AST and PATS data combined (b). The major nodes are indicated by the color green.

Minimum spanning tree (MST) analysis of the SS-O157 AST data yielded 6 nodes with the major node comprising 12 isolates (C-18, C-23, C-28, C-31, C-34, C-36, C-39, C-42, C-53, C-54, C-73, C-78) sharing the same AST profile (Figure 3(a)). However, integrating the PATS profiles with AST data in generating MST did not yield any specific correlation of PATS to AST, showing that phylogeny did not dictate antibiotic resistance-susceptibility of SS-O157 (Figure 3(b)).

### 3.4. Minimum Inhibitory Concentration (MIC) Testing Reliably Verified AST Results

Bacterial isolates (SS-O157, control O157, and bovine *E. coli*) with intermediate resistant (I) AST phenotype showed low levels of MIC (*μ*g/mL) to the respective antibiotics; MIC (*μ*g/mL) was less than or equal to the “susceptible range threshold” (Table 1). For instance, MIC (*μ*g/mL) was in the “susceptible range threshold” for six antibiotics (amoxicillin/clavulanic acid, ampicillin, cefoxitin, cefotaxime/ceftiofur, gentamicin, and nalidixic acid) in all isolates tested (Table 1).

One bovine *E. coli* isolate (912-4) had MIC in the “resistant range” for azithromycin (≥8 *μ*g/mL), sulfisoxazole (≥512 *μ*g/mL), and tetracycline (≥16 *μ*g/mL) corresponding to the resistant (R) AST phenotype observed with the same isolate (Table 1). Similarly, isolates with resistant (R)/intermediate resistant (I) AST phenotype with chloramphenicol (870-2) and streptomycin (870-2, C-104) had MIC above the resistance threshold (Table 1). In addition, three bovine *E. coli* (870-2, 888-2, 912-1) and three SS-O157 (C-66, C-87, C-104) isolates had MIC in the “resistant range” for sulfisoxazole, corresponding to the resistant (R) AST phenotype (Table 1). However, some of the isolates did not show high MIC for sulfisoxazole even if they had intermediate resistant (JEONG-1266, SS-52, EDL933, 914-3, C-2, C-5, C-13, C-14) or resistant (Sakai, 888-4, 912-2, C-4, C-11, C-12, C-18, C-22, C-38, C-45, C-49, C-80, C-85, C-88, C-90, C-99) AST phenotypes (Table 1). The cause for this discrepancy is unclear, however, given that the MIC results were obtained in a reproducible manner when done in triplicate makes it more reliable than the AST assay, which can be prone to batch-to-batch variation in antibiotic disks and human errors [64].

### 3.5. AR Associated Genes Were Amplified from SS-O157, Control O157, and Bovine *E. coli* Isolates

18/31 genes were amplified in at least one SS-0157 strain. Every tested isolate had >= 10 genes of which eight genes (*acrB, ais, arnA, macA, mdtH, yfbH, yjcP,* and *yjcR)* were present in all of the isolates tested (Tables 2 and [Supplementary-material supplementary-material-1]). AR specific genes detected in SS-O157 strains were *aac (3)-IV* (2 isolates: C7, C90), *sul*2 (3 isolates: C5, C99, C104), and *tetB* (1 isolate: C104) (Tables 2 and [Supplementary-material supplementary-material-1]). Overall, nine different PCR profiles based on the presence of genes associated with AR were identified in 53 SS-O157 isolates (Table 2). In addition, *int* that plays a role in horizontal gene transmission was amplified from 48/53 (91%) SS-O157 (Tables 2 and [Supplementary-material supplementary-material-1]).

One type of PCR profile, comprising 14 genes associated with AR (*acrB, ais, arnA, emrA, fsr, macA, marA, mdtH, mdtO, pmrD, rarD, yfbH, yjcP,* and *yjcR*) was prevalent among the 6 control O157 strains tested (Tables 2 and [Supplementary-material supplementary-material-1]). The *int* gene was present in all 6 control O157 strains as well (Tables 2 and [Supplementary-material supplementary-material-1]). Likewise, twelve genes (*acrB, ais, arnA, emrA, fsr, macA, mdtH, mdtO, rarD, yfbH, yjcP,* and *yjcR)* were present in all of the bovine *E. coli* isolates tested and all but one (888-1) contained the multiple antibiotic resistance operon (quinolone and tetracycline resistance) transcription factor, *marA* (Tables 3 and [Supplementary-material supplementary-material-1]). The streptomycin resistance gene (*aadA*1) was present in 15% of the isolates and *int* was present in 60% of the isolates. Polymyxin B resistance (*pmrD*) and sulfonamide resistance (*sul2*) were present in 85% and 10% and tetracycline resistance genes *tetA, tetB* and *tetC* were prevalent in 40, 20, and 25% of the bovine *E. coli* isolates (Tables 3 and [Supplementary-material supplementary-material-1]). Colistin resistance in bacteria from food producing animals is being actively researched [65], but no intermediate-resistant or resistant AST phenotypes against colistin was observed in this study and the *mcr1* and *mcr2* genes were not amplified.

### 3.6. Shiga Toxin Genes Were Amplified from SS-O157, Control O157, and Bovine *E. coli* Isolates

Primer pairs targeting Shiga toxin 1 and 2 genes (*stx1* and *stx2)* were used ([Supplementary-material supplementary-material-1]). The *stx1* gene was amplified from 49% of the SS-O157 isolates and the control O157 strains Sakai and EDL933 (Tables [Supplementary-material supplementary-material-1] and [Supplementary-material supplementary-material-1]). None of the bovine *E. coli* had the *stx1* gene ([Supplementary-material supplementary-material-1]). Except for one isolate, all SS-O157 isolates amplified the *stx2* gene ([Supplementary-material supplementary-material-1]). All control O157 strains and surprisingly four bovine *E. coli* isolates from one animal (4/20, 25%) amplified the *stx2* gene (Tables [Supplementary-material supplementary-material-1] and [Supplementary-material supplementary-material-1]). The Shiga toxin gene profiles of the SS-O157 isolates matched previously reported results [18].

### 3.7. Sequencing Results of Genes Associated with AR

PCR amplicons were sequenced in both directions and a consensus sequence was generated for 21 and 9 different AR genes from the SS-O157 and bovine *E. coli* isolates, respectively (Tables [Supplementary-material supplementary-material-1] and [Supplementary-material supplementary-material-1]). Amplicons targeting 16 AR genes (*acrB, ais, arnA, emrA, fsr, int, macA, marA, mdtH, mdtO, pmrD, rarD, tetB, yfbH, yjcP,* and *yjcR*) consistently aligned with their homologues following BLASTn analysis ([Supplementary-material supplementary-material-1]). Interestingly, amplicons for the *dfrA1, sul1, sul3, tetA* genes did not align with the respective homologues ([Supplementary-material supplementary-material-1]). Redesigned *tetA* primers ([Supplementary-material supplementary-material-1]) targeting the full length of *tetA* gene, also failed to amplify the full-length *tetA* gene from the control O157 and SS-O157 strains (data not shown). In contrast, the *tetA* gene was amplified with 99% identity to previously reported *tetA* genes upon BLASTn analysis from bovine *E. coli* ([Supplementary-material supplementary-material-1]).

Irrespective of the primer set used, nonspecific amplicons were obtained from some of the isolates when amplifying for the *mcr-*1 gene. The amplicons were gel purified and sequenced to confirm the genetic identity. The results confirmed the nonspecific amplification, and no homology to previously reported *mcr-*1 genes (GenBank) was observed (Tables [Supplementary-material supplementary-material-1] and [Supplementary-material supplementary-material-1]). The nonspecific amplification might have occurred due to the low specificity of primers and/or due to the presence of genes that have some sequence similarity to the *mcr-*1 gene (Tables [Supplementary-material supplementary-material-1] and [Supplementary-material supplementary-material-1]).

### 3.8. Sequencing Results of Shiga Toxin-2 Genes

Sequence analysis (Figures [Supplementary-material supplementary-material-1] and [Supplementary-material supplementary-material-1]) indicated that the *stx2* gene in the bovine *E. coli* isolates was similar (>97% identity) to the *stx2* gene in control O157 strains ([Supplementary-material supplementary-material-1] and [Supplementary-material supplementary-material-1]) although the latex agglutination test of all bovine *E. coli* (*n* = 20) against seven major STEC serogroups (O26, O45, O103, O111, O121, O145, and O157) was negative. Genetic distance (evolutionary divergence), based on the *stx2* sequences, within bovine *E. coli*, within control O157, and between bovine *E. coli* and control O157 isolates was 0.012, 0.006, and 0.038, respectively, indicating the subtle differences in the *stx2* sequences ([Supplementary-material supplementary-material-1], [Supplementary-material supplementary-material-1]). Neighbor joining and maximum likelihood phylogenetic trees showed distinct grouping and, therefore differences in the nucleotide sequence of *stx2* between control O157 and bovine *E. coli* isolates ([Supplementary-material supplementary-material-1]).

## 4. Discussion

In this study, aggregative adherence to RSE cells was found to be characteristic of all SS-O157 tested with 54% of the isolates demonstrating hyperadherence. SS-O157 isolates carried a minimal number of AR genes and some demonstrated phenotypic resistance to sulfisoxazole and tetracycline. Each SS-O157 isolate carried at least 10 genes directly or indirectly associated with AR, eight of which (*acrB, ais, arnA, macA, mdtH, yfbH, yjcP,* and *yjcR*) were present in all of the isolates. AR specific genes detected in SS-O157 strains were *aac (3)-IV* in 2 isolates, *sul*2 in 3 isolates, and *tetB* in one isolate. As reported previously [18] and determined using PATS in this study, the SS-O157 isolates were genetically diverse, but none of the adherence or AR profiles could be associated with a specific PATS type. However, PATS was able to cluster 94% of the SS-O157 with an identical PFGE pattern as that of human outbreak strains [18] into two clades, Clades 1 and 2, reflecting their possible distinction from other SS-O157 isolates tested.

Cattle and other food animals help to feed billions of people worldwide but can be reservoirs of infectious agents and antibiotic resistance [46, 65–67]. It has been 35 years since the first foodborne outbreak with O157 was reported [3], still the prevention and control of O157 infections continue to be a challenge due to complex factors affecting O157 colonization of cattle [66, 68–70] and the significant genetic diversity among O157 strains with only a subset being capable of causing human infections [45, 47]. Compared to the widely used bacterial fingerprinting technique, pulsed field gel electrophoresis (PFGE; 48), polymorphic amplified typing sequences (PATS) is a simple, user-friendly, easy-to-perform and interpret alternative that exploits both indels and SNPs in the bacterial genome to determine the genetic diversity of bacterial strains [50, 51]. In this study, we used PATS to categorize and ease the evaluation of 101 SS-O157 isolates. PATS assigned the SS-O157 isolates into five major clades, with the majority of the SS-O157 strains related to the human outbreak by their PFGE profiles [18] being grouped into two clades, Clade 1 and 2, clearly distinguishing them from the other SS-O157 [49–51].

The persistence of O157 in cattle has been shown to be potentially dependent on the adherence of bacteria to cells at the RAJ [71]. Significant intervention strategies to reduce O157 carriage and shedding in cattle can be developed by understanding the host-pathogen interactions at the rectoanal junction (RAJ) [52]. Rectoanal junction squamous epithelial (RSE) cell adherence assay, which mimics the natural process of colonization in cattle, has been successfully used to determine the adherence characteristics of O157, non-O157 STEC, and *Shigella* spp. [52–56]. RSE cell adherence assay has been used to identify the role of several proteins like the outer membrane protein A (OmpA) [72] and curli fimbriae [54], in modulating the colonization of O157 in cattle. Similar to other SS-O157 strains [19, 20], the 101 SS-O157 isolates evaluated in this study aggregatively adhered to bovine RSE cells with majority demonstrating hyperadherence; such strong adherence may contribute to their persistence in cattle. In contrast, SS-O157 strains exhibited diffuse adherence to HEp-2 cells with some strains not adhering at all. This observation confirmed previous reports that adherence to the commonly used non-GIT human cell line, HEp-2, does not reflect true bacterial interactions with human or bovine intestinal cells [52–56, 72] which is host dependent manner. A hyperadherent phenotype was observed on RSE cells with 69% of bovine SS-O157 strains whose PFGE pattern was identical to the human outbreak *E. coli* O157 strains on the CDC Pulse Net database [18] suggesting a possible link between increased bovine RAJ persistence and ensured contamination of meat/environment contributing to human infections [14–17]. These results highlight the importance of RSE cell adherence assay in studying O157 isolates and emphasizes the need to characterize factors that may contribute to the persistence and shedding of O157 by cattle.

It is hard to prevent or control STEC shedding by cattle because of widespread prevalence [63], presence of multiple virulence factors [73], pathogenicity [74], efficient survival mechanisms [75], and multiple factors influencing shedding [66, 70]. Efficacy of antibiotic treatment against STEC infections in humans is debatable [27], but antibiotics such as rifaximin, fosfomycin, azithromycin, and meropenem that do not stimulate the release of Shiga toxin from STEC are being suggested for the treatment of early stages of STEC disease to prevent HUS [23–27]. Although not currently favored, widespread antibiotic resistance in STEC isolated from cattle [76], environment [77], and humans [40] could preclude possible future use of such antibiotics to control STEC infections in humans [78]. In addition, although antibiotics are not used to clear STEC in cattle, the presence of AR genes in STEC could contribute towards increased dissemination of antibiotic resistance in the environment. Several studies in the US and abroad have reported resistance to aminoglycosides, *β*-lactams, carbapenems, cephalosporins, erythromycin, phenicols, streptomycin, sulpha-drugs, and tetracyclines, besides multidrug resistance in STEC isolated from humans and animals [31, 76, 79–82]. Meta-analysis of the AR data for the six control O157, as annotated in the Pathosystems Resource Integration Center (PATRIC) Bioinformatics Resource Center (https://www.patricbrc.org; [83]) also indicated the presence of ∼70 genes, directly or indirectly associated with AR. This genome data along with the published analysis of AR genes in STEC provided the impetus for determining the resistance profile of field SS-O157 isolates analyzed in the present study.

In our study, all the bacterial isolates tested had varied susceptibility to 17 antibiotics in AST assay, with the majority demonstrating resistance to sulfisoxazole. Resistance towards sulfisoxazole was detected both by AST and MIC in three SS-O157 isolates tested (C-66, C-87, C-104; Table 1) with PCR detection of only the *sul2* gene, which occurs as part of a variable resistance region on small, nonconjugative plasmids in *Enterobacteriaceae* members causing resistance against streptomycin and sulfisoxazole [84]. Sulfisoxazole resistance in *E. coli* isolates from swine fecal samples and other food animals has been associated with *sul1, sul2, sul3, dhfr,* and *dfrA1* genes [85, 86]. In another study, high MIC (>256 *μ*g/mL) for sulfisoxazole among *Salmonella spp.* isolates from retail meat, food animals, and humans was associated with the resistance genes *sul1, sul2,* and *sul3* [87], Resistant isolates from the AST assay had high MIC (*μ*g/mL), while intermediate resistant isolates were found to have MIC in the susceptible or intermediate range to the respective antibiotics. AR genes were amplified from the genomic DNA of some of the bacterial isolates tested with strain to strain variation among SS-O157 and bovine *E. coli* isolates; a uniform set of AR genes was amplified among six control O157 strains. The integron-integrase gene, *int*, linked to AR acquisition/transmission, was identified in 92% SS-O157 and 69% of the control O157 and bovine *E. coli* isolates [88, 89]. Since we evaluated only a limited number of genes associated with the phenotypic resistance against the respective antibiotic, we may have missed other genes that contribute to the same resistance and the lack of specificity of some primers may have further confounded this result. The *mcr-1* (either short or full length) gene was not identified in any of the bacterial isolates. Sequence analysis of AR genes from SS-O157 isolates indicated lower or no homology with the previously reported AR genes in NCBI database while the PCR amplicons from bovine *E. coli* isolates had high homology (>99% identity) with previously reported AR genes. Interestingly, we also identified *stx*2 genes in some *E. coli* isolates from cattle that were not homologous to *stx*2 in human outbreak O157 strains like EDL933, Sakai or EC4115 suggesting that these could be variant *stx*2. Since the *E. coli* were non-O157 and did not agglutinate with sera targeting the “big six” STEC serotypes O26, O45, O103, O111, O121, and O145, this result suggests that these were different STEC strains requiring further characterization.

## 5. Conclusion

In conclusion, our results indicate that SS-O157 could potentially persist longer at the bovine RAJ and harbor few AR genes providing limited resistance towards clinically useful antibiotics. Factors contributing to the aggregative adherence phenotype, the role of this adherence phenotype in bovine colonization and persistence, and the functional relevance of the AR associated genes in SS-O157 are presently being evaluated.

## Figures and Tables

**Table 1:** MIC profiles of all isolates with an intermediate to resistant phenotype in the AST assay.

Strains^c^	Description	MIC profile^a^ (*μ*g/mL) (using Etest® MIC strips^b^)										
		XL	AM	AZ	CL	CT	FX	GM	NA	SM	SX	TE
*E. coli* ATCC25922	Test control	8	4	7	8	0.09	3	0.38	3	2	64	2
Sakai	Control O157	6	3	4	8	0.09	6	0.25	4	3	128	3
EDL933	Control O157	4	3	4	8	0.08	8	0.25	4	2	64	3
EC4115	Control O157	S	S	1	6	S	3	S	S	S	S	3
JEONG-1266	Control SS-O157	6	3	4	8	0.06	6	0.38	4	6	128	2
SS-17	Control SS-O157	4	2	3	6	0.06	4	0.38	3	6	96	3
SS-52	Control SS-O157	6	3	3	8	0.08	6	0.38	6	6	96	3
870-1	*E. coli*	S	S	1	S	S	S	S	S	1	S	6
870-2	*E. coli*	8	4	0.75	256	S	S	S	S	384	1024	128
870-4	*E. coli*	3	2	4	4	0.04	2	0.38	4	1.5	48	6
888-1	*E. coli*	S	S	1.5	S	S	S	S	S	S	S	16
888-2	*E. coli*	6	3	3	8	0.09	6	0.25	6	6	1024	4
888-4	*E. coli*	S	S	1.5	S	S	S	S	S	1	128	S
912-1	*E. coli*	S	S	1.5	S	S	S	S	6	2	1024	S
912-2	*E. coli*	S	S	1.5	S	S	S	S	S	3	192	96
912-4	*E. coli*	3	2	8	8	0.03	3	0.38	4	8	1024	48
914-1	*E. coli*	S	S	2	S	S	4	S	S	1	S	4
914-2	*E. coli*	S	6	1	S	S	S	S	S	1	S	S
914-3	*E. coli*	8	8	2	S	S	S	S	S	1.5	128	S
914-4	*E. coli*	8	6	2	S	S	S	S	S	1.5	S	S
887-1	*E. coli* (STEC)	4	4	7	6	0.06	3	0.38	4	6	48	3
887-2	*E. coli* (STEC)	6	4	4	6	0.09	3	0.38	4	6	48	3
887-3	*E. coli* (STEC)	8	4	4	6	0.08	2	0.38	4	6	48	3
887-4	*E. coli* (STEC)	6	4	4	6	0.06	3	0.38	6	2	48	3
C-1	SS-O157	6	4	3	6	0.09	6	0.25	4	4	128	2
C-2	SS-O157	6	3	4	8	0.06	6	0.38	4	6	64	2
C-4	SS-O157	S	S	1	S	S	S	S	S	S	192	3
C-5	SS-O157	S	S	1	6	S	S	S	S	S	192	S
C-7	SS-O157	6	3	3	8	0.06	6	0.38	8	4	128	3
C-9	SS-O157	S	S	1	S	0.06	S	S	S	1.5	S	S
C-11	SS-O157	S	S	1	6	0.05	S	S	S	S	64	S
C-12	SS-O157	S	S	1	8	0.05	S	S	S	S	128	3
C-13	SS-O157	S	S	1	S	S	S	S	S	S	192	S
C-14	SS-O157	S	S	1	6	S	S	S	S	3	128	S
C-18	SS-O157	S	S	1.5	S	S	S	S	S	3	96	S
C-19	SS-O157	S	S	1.5	4	0.05	S	S	S	4	S	S
C-22	SS-O157	S	S	0.75	S	S	S	0.25	S	2	96	S
C-32	SS-O157	S	S	1	8	S	S	S	S	2	S	S
C-38	SS-O157	S	S	1	8	0.06	S	S	S	2	192	4
C-40	SS-O157	4	4	4	8	0.06	6	0.38	4	6	64	2
C-45	SS-O157	6	4	3	6	0.11	6	0.25	4	6	192	3
C-49	SS-O157	6	4	4	8	0.06	4	0.38	4	6	128	3
C-66	SS-O157	S	S	1	8	0.06	S	S	6	S	1024	S
C-77	SS-O157	6	3	3	6	0.06	4	0.5	4	8	48	3
C-80	SS-O157	6	3	3	8	0.06	6	0.25	8	0.75	64	3
C-85	SS-O157	S	S	1	S	0.05	S	S	S	S	256	S
C-87	SS-O157	S	S	1	S	0.05	S	S	S	1	1024	S
C-88	SS-O157	S	4	1	S	0.06	S	S	S	1	256	S
C-90	SS-O157	S	S	1	S	S	S	S	S	S	256	S
C-99	SS-O157	4	3	4	6	0.08	6	0.25	6	1.5	64	3
C-104	SS-O157	4	3	4	8	0.09	6	0.25	6	24	1024	256

^a^MIC Profile: MIC of 11 antibiotics was determined in all the isolates that were either Intermediate or Resistant on AST assay; remaining isolates are labelled S against the MIC strip to which the isolates were susceptible in AST assay. ^b^Antibiotic MIC Strips: XL = Amoxicillin/Clavulanic acid; AM = Ampicillin; AZ = Azithromycin; CL = Chloramphenicol; CT = Cefotaxime (in place of Ceftiofur); FX = Cefoxitin; GM = Gentamicin; NA = Nalidixic Acid; SM = Streptomycin; SX = Sulfisoxazole; TE = Tetracycline. ^c^Only one representative isolate was used for MIC testing if AST profile was the same.

**Table 2:** AR and integrase gene profiles of SS-O157 (*n* = 53) isolates and control O157 (*n* = 6) strains.

	O157 strain grouping	PCR profiles^a^
SS-O157 isolates	C1	*acrB, ais, arnA, emrA, fsr, int, macA, mdtH, pmrD, rarD, yfbH, yjcP, yjcR*
	C5	*acrB, ais, arnA, emrA, fsr, int, macA, marA, mdtH, mdtO, pmrD, rarD, sul2, yfbH, yjcP, yjcR*
	C2, 4, 6, 9, 11, 12, 13, 15, 17, 18, 19, 22, 28, 31, 32, 34, 35, 36, 38, 39, 40, 42, 53, 54, 57, 62, 66, 73, 74, 76, 77, 78, 80, 83, 85, 87, 88, 93, 94, 96, 98, 101	*acrB, ais, arnA, emrA, fsr,int, macA, marA, mdtH, mdtO, pmrD, rarD, yfbH, yjcP, yjcR*
	C7, 90	*aac (3)-IV, acrB, ais, arnA, emrA, fsr, int, macA, marA, mdtH, mdtO, pmrD, rarD, yfbH, yjcP, yjcR*
	C14, 16	*acrB, ais, arnA, macA, marA, mdtH, mdtO, yfbH, yjcP, yjcR*
	C23, 49	*acrB, ais, arnA, macA, marA, mdtH, mdtO, pmrD, rarD, yfbH, yjcP, yjcR*
	C45	*acrB, ais, arnA, emrA, fsr, int, macA, marA, mdtH, pmrD, rarD, yfbH, yjcP, yjcR*
	C99	*acrB, ais, arnA, emrA, fsr, macA, marA, mdtH, mdtO, pmrD, rarD, sul2, yfbH, yjcP, yjcR*
	C104	*acrB, ais, arnA, emrA, fsr, int, macA, marA, mdtH, mdtO, pmrD, rarD, sul2, tetB, yfbH, yjcP, yjcR*
Control O157 strains	Sakai, EDL 933, JEONG-1266, SS 52	*acrB, ais, arnA, emrA, fsr, int, macA, marA, mdtH, mdtO, pmrD, rarD, sul2, yfbH, yjcP, yjcR*
	EC4115, SS 17	*acrB, ais, arnA, emrA, fsr, int, macA, marA, mdtH, mdtO, pmrD, rarD, yfbH, yjcP, yjcR*

^a^Genes: *aac(3)-IV* = Aminoglycoside resistance; *aadA1* = Streptomycin resistance; *acrB* *=* Acriflavine, aminoglycoside, and multidrug resistance efflux pump; *ais* = Polymyxin resistance protein, histidine phosphatase family protein; *arnA* = Polymyxin resistance protein; *bla*_*CTX-M*_ *=* Cephalosporin resistance; *bla*_*TEM*_ = Ampicillin resistance; *catA1* = Chloramphenicol resistance; *dfrA1* = Trimethoprim resistance; *dhfrI* = Trimethoprim resistance; *emrA* = Multidrug resistance protein A; *fsr* = Fosfomycin resistance; *int* = Integrase; *macA* = Macrolide-specific efflux protein; *marA* = Multiple antibiotic resistance protein; *mcr1* = Colistin resistance; *mcr2* = Colistin resistance; *mdtH* = Multidrug resistance protein; *mdtO* = Multidrug resistance protein; *mph(A)* = Macrolides resistance; *pmrD* = Polymyxin B resistance; *qnrA* = Quinolones resistance; *rarD* = Chloramphenicol resistance; *sul1* *=* Sulfonamide resistance; *tetA*, *tetB*, *tetC* = Tetracycline resistance; *yfbH* = Polymyxin resistance; *yjcP* = Outer membrane component of tripartite multidrug resistance system; *yjcR* = Inner membrane component of tripartite multidrug resistance system.

**Table 3:** AR and integrase gene profiles of 20 bovine *E. coli* isolates.

*E. coli* isolates	PCR profiles^a^
870-1	*acrB, ais, arnA, emrA, fsr, macA, marA, mdtH, mdtO, pmrD, rarD, tetC, yfbH, yjcP, yjcR*
870-2, 870-3	*acrB, ais, arnA, emrA, fsr, macA, marA, mdtH, mdtO, pmrD, rarD, sul2, tetA, yfbH, yjcP, yjcR*
870-4, 888-3	*acrB, ais, arnA, emrA, fsr, macA, marA, mdtH, mdtO, pmrD, rarD, tetA, tetC, yfbH, yjcP, yjcR*
887-1, 887-2, 887-3, 887-4, 888-2, 914-3, 914-4	*acrB, ais, arnA, emrA, fsr, int, macA, marA, mdtH, mdtO, pmrD, rarD, yfbH, yjcP, yjcR*
888-1	*acrB, ais, arnA, emrA, fsr, macA, mdtH, mdtO, pmrD, rarD, tetA, tetC, yfbH, yjcP, yjcR*
888-4	*acrB, ais, arnA, emrA, fsr, macA, marA, mdtH, mdtO, pmrD, rarD, yfbH, yjcP, yjcR*
912-1	*aadA1, acrB, ais, arnA, emrA, fsr, int, macA, marA, mdtH, mdtO, rarD, tetA, tetB, tetC, yfbH, yjcP, yjcR*
912-2	*aadA1, acrB, ais, arnA, emrA, fsr, int, macA, marA, mdtH, mdtO, rarD, tetB, yfbH, yjcP, yjcR*
912-3	*acrB, ais, arnA, emrA, fsr, macA, marA, mdtH, mdtO, pmrD, rarD, tetA, yfbH, yjcP, yjcR*
912-4	*aadA1, acrB, ais, arnA, emrA, fsr, int, macA, marA, mdtH, mdtO, rarD, tetB, yfbH, yjcP, yjcR*
914-1	*acrB, ais, arnA, emrA, fsr, int, macA, marA, mdtH, mdtO, pmrD, rarD, tetB, yfbH, yjcP, yjcR*
914-2	*acrB, ais, arnA, emrA, fsr, int, macA, marA, mdtH, mdtO, pmrD, rarD, tetA, yfbH, yjcP, yjcR*

^a^Genes: *aac(3)-IV* = Aminoglycoside resistance; *aadA1* = Streptomycin resistance; *acrB* *=* Acriflavine, aminoglycoside, and multidrug resistance efflux pump; *ais* = Polymyxin resistance protein, histidine phosphatase family protein; *arnA* = Polymyxin resistance protein; *bla*_*CTX-M*_ *=* Cephalosporin resistance; *bla*_*TEM*_ = Ampicillin resistance; *catA1* = Chloramphenicol resistance; *dfrA1* = Trimethoprim resistance; *dhfrI* = Trimethoprim resistance; *emrA* = Multidrug resistance protein A; *fsr* = Fosfomycin resistance; *int* = Integrase; *macA* = Macrolide-specific efflux protein; *marA* = Multiple antibiotic resistance protein; *mcr1* = Colistin resistance; *mcr2* = Colistin resistance; *mdtH* = Multidrug resistance protein; *mdtO* = Multidrug resistance protein; *mph(A)* = Macrolides resistance; *pmrD* = Polymyxin B resistance; *qnrA* = Quinolones resistance; *rarD* = Chloramphenicol resistance; *sul1* *=* Sulfonamide resistance; *tetA*, *tetB*, *tetC* = Tetracycline resistance; *yfbH* = Polymyxin resistance; *yjcP* = Outer membrane component of tripartite multidrug resistance system; *yjcR* = Inner membrane component of tripartite multidrug resistance system.

## Data Availability

All data generated or analyzed during this study are included in this published article (and its supplementary information files).

## Supplementary Materials

Supplementary Materials The supplementary files contain the supplementary tables supporting the results in the manuscript, and as listed in the same, include the following: Table S1: zone of inhibition diameters used to determine AST profiles of control *Escherichia coli* (ATCC®25922™) and all bacterial isolates tested in this assay. Table S2: CLSI break points for determining MIC (*μ*g/mL) profiles of control *Escherichia coli* (ATCC®25922™) and all bacterial isolates tested in this assay. Table S3: primers used in this study. Table S4: PATS profiles of SS-O157 (*n* = 101) isolates. Table S5: adherence patterns of SS-O157 (*n* = 53) isolates and control O157 (*n* = 6) strains on RSE cells. Table S6: adherence patterns of SS-O157 (*n* = 53) isolates and control O157 (*n* = 6) strains on HEp-2 cells. Table S7: AST profiles of 53 SS-O157 isolates against 17 antibiotics. Table S8: AST profiles of six control O157 strains against 17 antibiotics. Table S9: AST profiles of 20 bovine *E. coli* isolates against 17 antibiotics. Table S10: AR and integrase gene profiles of SS-O157 (*n* = 53) isolates. Table S11: AR and integrase gene profiles of control O157 (*n* = 6) strains. Table S12: AR and integrase gene profiles of 20 bovine *E. coli* isolates. Table S13: AR and integrase gene sequencing results for representative SS-O157 isolates. Table S14: AR gene sequencing results for representative bovine *E. coli* isolates and control O157 strains. Table S15: Stx2 gene: estimates of evolutionary divergence over sequence pairs between two groups and within each group. Figure S1: AST (panel A) and MIC (panel B) testing is shown for a representative SS-O157 strain C-104 against ampicillin and tetracycline. Figure S2: nucleotide (A) and amino acid (B) sequence of the Stx2 genes amplified and sequenced from six control O157 and four bovine *E. coli* isolates. Sequences were aligned using ClustalW, multiple sequence alignment program. Figure S3: neighbor joining (A) and maximum likelihood (B) phylogenetic trees of Stx2 gene sequences from six control O157 and four bovine *E. coli* isolates. Variation in nucleotides along the Stx2 gene sequences may explain subtle differences between bovine *E. coli* and control O157 isolate groups.
