# Supplementary material for: Supershed Escherichia coli O157:H7 Has Potential for Increased Persistence on the Rectoanal Junction Squamous Epithelial Cells and Antibiotic Resistance
Source: Int J Microbiol. 2020 Apr 13;2020:2368154. doi: 10.1155/2020/2368154 (PMC7178529; doi:10.1155/2020/2368154)
Supplement: Supplementary Materials — The supplementary files contain the supplementary tables supporting the results in the manuscript, and as listed in the same, include the following: Table S1: zone of inhibition diameters used to determine AST profiles of control Escherichia coli (ATCC®25922™) and all bacterial isolates tested in this assay. Table S2: CLSI break points for determining MIC (μg/mL) profiles of control Escherichia coli (ATCC®25922™) and all bacterial isolates tested in this assay. Table S3: primers used in this study. Table S4: PATS profiles of SS-O157 (n = 101) isolates. Table S5: adherence patterns of SS-O157 (n = 53) isolates and control O157 (n = 6) strains on RSE cells. Table S6: adherence patterns of SS-O157 (n = 53) isolates and control O157 (n = 6) strains on HEp-2 cells. Table S7: AST profiles of 53 SS-O157 isolates against 17 antibiotics. Table S8: AST profiles of six control O157 strains against 17 antibiotics. Table S9: AST profiles of 20 bovine E. coli isolates against 17 antibiotics. Table S10: AR and integrase gene profiles of SS-O157 (n = 53) isolates. Table S11: AR and integrase gene profiles of control O157 (n = 6) strains. Table S12: AR and integrase gene profiles of 20 bovine E. coli isolates. Table S13: AR and integrase gene sequencing results for representative SS-O157 isolates. Table S14: AR gene sequencing results for representative bovine E. coli isolates and control O157 strains. Table S15: Stx2 gene: estimates of evolutionary divergence over sequence pairs between two groups and within each group. Figure S1: AST (panel A) and MIC (panel B) testing is shown for a representative SS-O157 strain C-104 against ampicillin and tetracycline. Figure S2: nucleotide (A) and amino acid (B) sequence of the Stx2 genes amplified and sequenced from six control O157 and four bovine E. coli isolates. Sequences were aligned using ClustalW, multiple sequence alignment program. Figure S3: neighbor joining (A) and maximum likelihood (B) phylogenetic trees of Stx2 gene sequences fr [file 2368154.f1.zip › 2368154.f1/mat.2368154.v2.pdf]

Table S1. Zone of inhibition diameters (mm) used to determine AST profiles of control *Escherichia coli* (ATCC®25922™) and all bacterial isolates tested in this assay.

Antibiotic	Disc Code	Antibiotic Concentration (µg)	Control Strain Zone Diameter (mm)	Test Zone diameters (mm)		
				Resistant	Intermediate	Susceptible
Amoxicillin/Clavulanic acid	AmC-30	20/10	18—24	<= 13	14—17	>= 18
Ampicillin	AM-10	10	16—22	<= 13	14—16	>= 17
Azithromycin	AZM-15	15	14—21	<= 13	14—17	>= 18
Cefoxitin	FOX-30	30	23—29	<= 14	15—17	>= 18
Ceftiofur	XNL-30	30	26—31	<= 14	15—20	>= 21
Ceftriaxone	CRO-30	30	29—35	<= 13	14—20	>= 21
Chloramphenicol	C-30	30	21—27	<= 12	13—17	>= 18
Ciprofloxacin	CIP-5	5	30—40	<= 15	16—20	>= 21
Colistin	CL-10	10	11—15	<= 08	09—10	>= 11
Fosfomycin	FOS200	200	22—30	<= 12	13—15	>= 16
Gentamicin	GM-10	10	19—26	<= 12	13—14	>= 15
Nalidixic Acid	NA-30	30	22—28	<= 13	14—18	>= 19
Polymyxin B (Units)	PB-300	300	13—19	<= 08	09—11	>= 12
Streptomycin	S-10	10	12—20	<= 11	12—14	>= 15
Sulfamethoxazole/Trimethoprim	SXT	23.75/1.25	23—29	<= 10	11—15	>= 16
Sulfisoxazole	G-.25	0.25	15—23	<= 12	13—16	>= 17
Tetracycline	TE-30	30	18—25	<= 14	15—18	>= 19

[a] Adapted from M100, Performance Standards for Antimicrobial Susceptibility Testing; 27th Edition, (Published by Clinical and Laboratory Standards Institute, CLSI 2017, Pennsylvania, USA)

Table S2. CLSI break points for determining MIC (µg/mL) profiles of control *Escherichia coli* (ATCC®25922™) and all bacterial isolates tested in this assay.

MIC Strips (Biomereiux, E-test®)	Strip Code (E-test)	Antibiotic Concentration (µg)	Control Strain MIC Range (µg/mL)	Interpretive Breakpoints for test *E. coli* MIC (µg/mL)	
				Susceptible	Resistant
Amoxicillin/ Clavulanic acid	XL	0.016-256 µg	2/1--8/4	<= 8/4	>= 32/16
Ampicillin	AM	0.016-256 µg	2—8	<=8	>=32
Azithromycin [b]	AZ	0.016-256 µg	0.5—2	<=2	>=8
Cefoxitin	FX	0.016-256 µg	2—8	<=8	>=32
Ceftiofur/Cefotaxime	CT	0.002- 32 µg	0.03--0.12	<=2	>=8
Ceftriaxone	TX	0.016-256 µg	0.03--0.12	<=8	>= 64
Chloramphenicol	CL	0.016-256 µg	2—8	<=8	>=32
Ciprofloxacin	CI	0.002- 32 µg	0.004--0.015	<= 0.06	>= 0.12
Colistin [c]	CO	0.016-256 µg	0.5 – 4	<=2	>=8
Fosfomycin	FM	0.064-1024 µg	0.5 -- 2.0	<= 64	>= 256
Gentamicin	GM	0.016-256 µg	0.25 -- 1.0	<=4	>= 8
Nalidixic Acid	NA	0.016-256 µg	1 – 4	<=8	>=32
Polymyxin B [c]	PO	0.064-1024 µg	0.25 -- 2.0	<=2	>=8
Streptomycin [d]	SM	0.064-1024 µg	8 – 32	<= 4	>=16
Trimethoprim/Sulfamethoxazole	TS	0.016-256 µg	<= 0.5/9.5	<= 2/38	>= 4/76
Sulfisoxazole	SX	0.064-1024 µg	8 – 32	<= 256	>= 512
Tetracycline	TE	0.016-256 µg	0.5 -- 2.0	<=4	>=16

[a] Adapted from M100, Performance Standards for Antimicrobial Susceptibility Testing; 27th Edition, (Published by Clinical and Laboratory Standards Institute, CLSI 2017, Pennsylvania, USA)

[b] The acceptable range for *Staphylococcus aureus* ATCC®25923™ against Azithromycin have been shown

[c] The acceptable range for *Pseudomonas aeruginosa* ATCC®27853™ against Colistin and Polymyxin B have been shown

[d] Breakpoints for *Yersinia pestis*; plates have to be incubated for 24 hours before reading the MIC of Streptomycin in *E. coli* isolates

Table S3. Primers used in this study

Genes[a]	Forward	Reverse	Amplicon Size (bp)	Tm	Reference/Gene[b]
aac(3)-IV	CTTCAGGATGGCAAGTTGGT	TCATCTCGTTCTCCGCTCAT	286	58	90
aadA1	TATCCAGCTAAGCGCGAACT	ATTTGCCGACTACCTTGGTC	447	58	90
acrB	GTTGTTCCATCAGCTCCATT	GACCAGGGCGTATTTATGAC	838	56	Accession # LR134031.1
Ais	CCACATGCATGACTAAACCA	TCGTTCAACCAATCAATGCT	362	56	Accession # CP037941.1
arnA	GTTTAACTGGATGGGACCAC	GTTCTCAGGATTGCCAATGT	241	56	Accession # LR134092.1
$bla_{CTX\text{-}M}$	TTTGCGATGTGCAGTACCAGTAA	CGATATCGTTGGTGGTGCCATA	544	51	91
bla_{TEM}	GAGTATTCAACATTTTCGT	ACCAATGCTTAATCAGTGA	857	58	90
catA1	AGTTGCTCAATGTACCTATAACC	TTGTAATTCATTAAGCATTCTGCC	547	55	90
dfrA1	GGAGTGCCAAAGGTGAACAGC	GAGGCGAAGTCTTGGGTAAAAAC	367	50	92
dhfrI	AAGAATGGAGTTATCGGGAATG	GGGTAAAAACTGGCCTAAAATTG	391	58	90
emrA	GAAGAAACCGATGACGCATA	GGTTAGTGGTATTGACGCTC	901	56	Accession # CP020495.1
Fsr	ATCAGGCAGGAATATGGTCA	TTTTTCGGTTTTGCTTTCGG	141	56	Accession # LR134092.1
int (Integrase)	TGCTGGTACATCCTAATGGT	CAGGCGAGAAAGATCAAGAG	545	56	Accession # AP018488.1
macA	GTGATTTACAGCAAAGCGTG	CAGCACATTTTTCACATCGG	791	56	Accession # CP020495.1
marA	GGGTTACTCTAAATGGCACC	GTATCGGCTCGTTACTTTCC	128	56	Accession # CP035366.1
mcr1	CGTATCATTGGTTTGGGTGT	GATCAGCATATCTTTGCCGT	780	56	Accession # CP035313.1
mcr1	GCTGATTTTACTGCCTGTGG	TACGCATATCAGGCTTGGTT	250	55	Drs. Ricker & Allen
mcr1(full length)	ATGATGCAGCATACTTCTGTG	TCAGCGGATGAATGCGGTG	1626	60	43
mcr2	TGGTACAGCCCCTTTATT	GCTTGAGATTGGGTTATGA	538	56	93
mdtH	GCTAAACCCCATATAGCTGC	AGCGTTTTGTCACCTATGTC	392	56	Accession # LR134092.1
mdtO	GCCTGAGAATCAGCAAAAGA	GTATCGTTGTTGGCCTCTAC	328	56	Accession # CP020495.1
mph(A)	AACTGTACGC ACTTGC	GGTACTCTTCGTTACC	837	52	94
pmrD	TTTTCCCTGCCACTTTACAC	AAAATGATCGCCGAAGTGAA	175	56	Accession # LR134092.1
qnrA	GGGTATGGATATTATTGATAAAG	CTAATCCGGCAGCACTATTTA	670	50	95
rarD	GCCGTAGAAGGCAAAACTAA	CGTGATCTGGTCGTTTTTCT	370	56	Accession # BA000007.3
*stx*1 (*Shiga toxin* 1)	ATAAATCGCCATTCGT TGACTAC	AGAACGCCCACTGAGATCATC	180	61	96
*stx*2 (*Shiga toxin* 2)	GGCACTGTCTGAAACTGCTCC	TCGCCAGTTATCTGACAT TCTG	255	65	96
sul1	CCAAGGATTTCCTGACCCTG	GGGTTTCCGAGAAGGTGATT	479	58	Accession # MH765658.1
sul2	GCTCATCATTTTCGGCATCG	TTCGCCAATTGCGGATAGAA	348	58	Accession # MH765656.1
sul3	TGGTGTAAAGCGTGAACGAA	TTAACATCATGGGTGCGGAG	264	58	Accession # MH765654.1
tetA	GGCCTCAATTTCCTGACG	AAGCAGGATGTAGCCTGTGC	372	59	97
tetA (full length)	GTGAAACCCAACAGACCCCTGA	CCGTTCCACGTTGTTATAGAAGC	1103	61	Accession # MK461931.1
tetB	GAGACGCAATCGAATTCGG	TTTAGTGGCTATTCTTCCTGCC	228	60	97
tetC	TGCTCAACGGCCTCAACC	AGCAAGACGTAGCCCAGCG	379	63	97
yfbH	GTGAAGCCACAGTTTTTGTG	ATGAATGGTATAAACCGGCG	543	56	Accession # CP020495.1
yjcP	GGTAATGCCACTGGTTTGAT	TTCACAATTACAGGTCGCAG	540	56	Accession # CP035366.1
yjcR	TAGAACGAGAAACTTTCGGC	AGCAAATTATGCTCACCCAG	621	56	Accession # CP035366.1

[a] AR genes: *aac(3)-IV* = Aminoglycoside resistance; *aadA1* = Streptomycin resistance; *acrB* = Acriflavine, aminoglycoside and multidrug resistance efflux pump; *ais* =Polymyxin resistance protein, histidine phosphatase family protein; *arnA* = Polymyxin resistance protein; bla_{CTX-M} = Cephalosporin resistance; bla_{TEM} = Ampicillin resistance; *catA1* = Chloramphenicol resistance; *dfrA1* = Trimethoprim resistance; *dhfrI* = Trimethoprim resistance; *emrA* = Multidrug resistance protein A; *fsr* = Fosfomycin resistance; *macA* = Macrolide-specific efflux protein; *marA* = Multiple antibiotic resistance protein; *mcr1* = Colistin resistance; *mcr2* = Colistin resistance; *mdtH* = Multidrug resistance protein; *mdtO* = Multidrug resistance protein; *mph(A)* = Macrolides resistance; *pmrD* = Polymyxin B resistance; *qnrA* = Quinolones resistance; *rarD* = Chloramphenicol resistance; *sul1*= Sulfonamide resistance; *tetA*, *tetB*, *tetC* = Tetracycline resistance; *yfbH* = Polymyxin resistance; *yjcP* = Outer membrane component of tripartite multidrug resistance system; *yjcR* = Inner membrane component of tripartite multidrug resistance system

[b] Primers were derived either from those reported in the literature or from gene sequences available in GenBank.

Table S4. PATS profiles of SS-O157 (n=101) isolates.

SS-O157	Polymorphic *Xba*I sites[a]								Polymorphic *Avr*II sites[a]							Virulence Genes[a]			
	IK8	IK25	IK114	IK118	IK123	IK127	IKB3	IKB5	IKNR3	IKNR7	IKNR10	IKNR12	IKNR16	IKNR27	IKNR33	*stx*1	*stx*2	*eae*	*hly*A
C1	0	0	1	1	1	0	0	0	0	2	2	2	1	2	2	0	1	1	1
C2	1	0	0	1	1	1	0	0	1	2	2	2	1	0	2	0	1	1	1
C3	1	0	1	0	1	1	0	1	2	2	2	2	2	2	2	1	1	1	1
C4	0	0	1	1	0	1	0	1	2	2	2	0	2	2	2	1	1	1	1
C5	0	0	1	1	1	1	1	0	2	2	2	2	1	2	2	0	1	1	1
C6	0	0	0	1	1	1	1	0	2	2	2	2	1	2	2	0	1	1	1
C7	0	0	1	1	1	1	1	0	2	2	2	2	1	2	2	0	1	1	1
C9	1	0	0	1	1	1	0	0	1	0	2	0	1	2	0	1	0	1	1
C11	1	0	1	1	1	1	1	1	2	0	2	0	2	2	2	1	1	1	1
C16	0	0	0	1	1	1	0	0	0	0	2	0	1	2	2	0	1	1	1
C17	1	0	1	1	1	1	0	0	2	0	2	0	1	2	2	0	1	1	0
C22	1	0	1	1	1	1	1	0	2	2	2	2	1	0	2	0	1	1	1
C25	0	0	1	1	1	1	0	0	0	2	2	2	1	2	2	1	1	1	1
C26	1	0	1	1	1	1	0	1	2	1	1	2	2	2	2	1	1	1	1
C27	0	0	1	1	0	1	0	1	2	2	2	2	2	2	2	1	1	1	1
C31	0	0	1	1	1	1	1	1	2	2	2	2	1	2	2	0	1	1	1
C37	1	0	1	1	1	1	0	1	2	2	2	2	2	2	2	0	1	1	1
C39	1	0	1	1	1	1	1	1	2	2	2	2	1	2	2	0	1	1	1
C44	1	0	1	1	1	0	0	0	1	2	2	2	1	2	2	1	1	1	0
C46	0	1	1	1	0	0	0	0	0	2	2	2	1	2	2	0	1	1	1
C48	1	0	0	1	0	1	0	1	1	2	2	2	1	2	0	0	1	1	1
C50	1	0	1	1	0	1	0	1	2	2	2	2	2	2	2	1	1	1	1
C55	1	0	1	0	1	1	0	0	2	2	2	2	1	2	2	0	1	1	1
C56	1	0	1	0	1	1	0	0	1	2	2	2	1	2	2	0	1	1	1
C59	1	0	0	1	1	1	0	0	0	2	2	2	1	2	0	0	1	1	1
C64	1	0	1	1	1	0	1	1	2	2	2	2	2	2	2	1	1	1	1
C68	1	0	1	1	1	1	0	0	2	2	2	2	2	2	2	1	1	1	1
C69	1	0	1	1	1	1	0	1	2	0	2	2	2	2	2	1	1	1	1
C70	1	0	0	1	1	0	0	0	1	2	2	2	1	2	0	1	0	1	1
C72	1	0	0	1	1	1	0	0	1	2	2	2	1	2	0	0	1	0	1

C73	1	1	1	1	1	1	1	0	2	2	2	2	1	2	2	0	1	1	1
C75	0	0	1	1	1	1	1	0	2	2	2	2	1	2	2	1	1	1	1
C76	0	0	1	1	1	1	1	0	2	0	2	2	1	2	2	0	1	1	1
C79	0	0	0	1	1	0	1	0	2	2	2	2	0	2	0	1	1	1	1
C80	1	0	1	0	1	1	0	1	1	2	2	2	1	2	2	0	1	1	1
C81	1	0	1	1	1	1	0	1	1	2	2	2	1	2	2	1	1	1	1
C82	1	0	0	1	1	1	0	0	1	0	2	0	1	2	0	1	1	1	1
C88	1	0	1	1	1	1	0	1	2	0	2	0	2	2	2	0	1	1	1
C89	1	0	1	1	1	1	1	0	2	2	0	2	1	2	2	0	1	1	1
C92	1	0	0	1	1	1	0	0	1	2	2	2	1	1	0	1	1	1	1
C95	1	1	0	1	1	1	0	0	1	2	2	2	1	2	0	1	1	1	1
C96	1	0	0	1	0	1	0	0	1	2	2	2	0	2	0	1	1	1	1
C97	1	0	1	1	1	1	1	1	2	0	0	2	2	2	2	1	1	0	1
C98	1	1	1	1	1	1	0	1	2	2	2	2	2	2	2	1	1	1	1
C101	1	0	0	1	1	1	0	1	2	2	2	2	2	2	2	1	1	1	1
C102	1	0	0	1	1	1	1	0	1	2	2	2	1	2	0	1	1	1	1
C104	1	0	1	1	1	1	1	1	1	2	2	2	1	2	2	1	1	1	1
C40, C41, C94	1	0	0	1	1	0	0	0	1	2	2	2	1	2	0	1	1	1	1
C28, C62, C67, C71, C85	1	0	0	1	1	1	0	0	1	2	2	2	1	2	0	0	1	1	1
C19, C20, C21, C57, C60, C83, C84, C91, C93	1	0	0	1	1	1	0	0	1	2	2	2	1	2	0	1	1	1	1
C45, C47	1	0	1	1	1	0	0	0	0	2	2	2	1	2	2	0	1	1	1
C18, C49, C78	1	0	1	1	1	0	0	0	2	2	2	2	1	2	2	0	1	1	1
C61, C77	1	0	1	1	1	1	0	0	2	1	1	2	1	2	2	0	1	1	1
C8, C23, C24, C33, C43, C86, C87, C90, C100, C103	1	0	1	1	1	1	0	1	2	2	2	2	2	2	2	1	1	1	1
C51, C53	1	0	1	1	1	1	1	0	1	2	2	2	1	2	2	0	1	1	1
C14, C15, C29, C32, C34, C35, C36, C38, C42, C54, C63, C74	1	0	1	1	1	1	1	0	2	2	2	2	1	2	2	0	1	1	1
C12, C13, C30, C65, C66, C99	1	0	1	1	1	1	1	1	2	2	2	2	2	2	2	1	1	1	1

[a] 0: no amplicon; 1: amplicon with one *Xba*I or without *Avr*II site; 2: amplicon with a functional AvrII site

Table S5. Adherence patterns of SS-O157 (n=53) isolates and control O157 (n=6) strains on RSE cells.

Strains	Bacterial Adherence Pattern	Eukaryotic cells with adherent bacteria, in the ranges shown, for two different trials[1] (MOI[2] = 10^6 bacteria:10^5 cells)				Percent Mean +/- standard error of mean, of eukaryotic cells with adherent bacteria in the ranges shown[5]	
		Trial I		Trial II			
		>10	1-10[(3)]	>10	1-10	>10	1-10
C1	Aggregative, Moderate	23 (80)	57 (80)	13 (80)	67 (80)	22.5 ± 6.5	**77.5 ± 6.5**
C2	Aggregative, Strong	70 (80)	10 (80)	69 (80)	11 (80)	**86.75 ± 0.75**	13.25 ± 0.75
C4	Aggregative, Moderate	2 (80)	78 (80)	9 (80)	71 (80)	6.75 ± 4.25	**93.25 ± 4.25**
C5	Aggregative, Strong	77 (80)	3 (80)	76 (80)	4 (80)	**95.63 ± 0.625**	4.375 ± 0.625
C6	Aggregative, Strong	41 (80)	39 (80)	43 (80)	37 (80)	**52.5 ± 1.5**	47.5 + 1.5
C7	Aggregative, Strong	80 (80)	0 (80)	78 (80)	2 (80)	**98.75 ± 1.25**	1.25 ± 1.25
C9	Aggregative, Moderate	0 (80)	80 (80)	1 (80)	79 (80)	0.625 ± 0.625	**99.38 ± 0.625**
C11	Aggregative, Moderate	40 (80)	40 (80)	28 (80)	52 (80)	42.5 ± 7.5	**57.5 ± 7.5**
C12	Aggregative, Strong	80 (80)	0 (80)	51 (80)	29 (80)	**82 ± 18**	18 ± 18
C13	Aggregative, Strong	63 (80)	17 (80)	56 (80)	24 (80)	**74.5 ± 4.5**	25.5 ± 4.5
C14	Aggregative, Strong	31 (80)	49 (80)	76 (80)	4 (80)	**67 + 28**	33 + 28
C15	Aggregative, Strong	65 (80)	15 (80)	63 (80)	17 (80)	**80 ± 1**	20 ± 1
C16	Aggregative, Strong	40 (80)	40 (80)	60 (80)	20 (80)	**62.5 ± 12.5**	37.5 ± 12.57
C17	Aggregative, Strong	60 (80)	20 (80)	57 (80)	23 (80)	**73 ± 2**	27 ± 2
C18	Aggregative, Strong	41 (80)	39 (80)	50 (80)	30 (80)	**56.75 ± 5.75**	43.25 ± 5.75
C22	Aggregative, Moderate	28 (80)	51 (80)	0 (80)	80 (80)	17.5 ± 17.5	**82 ± 18**
C23	Aggregative, Moderate	28 (80)	52 (80)	24 (80)	56 (80)	32.5 ± 2.5	**67.5 ± 2.5**
C28	Aggregative, Moderate	21 (80)	59 (80)	30 (80)	40 (80)	32 ± 6	**62 ± 12**
C31	Aggregative, Moderate	14 (80)	66 (80)	14 (80)	66 (80)	17.5 ± 0	**82.5 ± 0**
C32	Aggregative, Strong	80 (80)	0 (80)	80 (80)	0 (80)	**100 ± 0**	0 ± 0

C34	Aggregative, Strong	75 (80)	5 (80)	77 (80)	3 (80)	**95.13 ± 1.125**	4.875 ± 1.125
C35	Aggregative, Strong	80 (80)	0 (80)	80 (80)	0 (80)	**100 ± 0**	0 + 0
C36	Aggregative, Strong	80 (80)	0 (80)	80 (80)	0 (80)	**100 ± 0**	0 + 0
C38	Aggregative, Strong	80 (80)	0 (80)	80 (80)	0 (80)	**100 ± 0**	0 + 0
C39	Aggregative, Strong	80 (80)	0 (80)	80 (80)	0 (80)	**100 ± 0**	0 ± 0
C40	Aggregative, Strong	80 (80)	0 (80)	77 (80)	3 (80)	**98.13 ± 1.875**	1.875 ± 1.875
C42	Aggregative, Strong	80 (80)	0 (80)	80 (80)	0 (80)	**100 ± 0**	0 ± 0
C45	Aggregative, Moderate	45 (80)	35 (80)	15 (80)	64 (80)	37.5 ± 18.5	**62 ± 18**
C49	Aggregative, Strong	69 (80)	11 (80)	74 (80)	6 (80)	**89.25 ± 3.25**	10.75 ± 3.25
C53	Aggregative, Moderate	23 (80)	55 (80)	23 (80)	53 (80)	29 ± 0	**67.5 ± 1.5**
C54	Aggregative, Strong	76 (80)	4 (80)	80 (80)	0 (80)	**97.5 ± 2.5**	2.5 ± 2.5
C57	Aggregative, Strong	43 (80)	37 (80)	40 (80)	40 (80)	**52 ± 2**	48 ± 2
C62	Aggregative, Strong	74 (80)	6 (80)	59 (80)	11 (80)	**83.25 ± 9.25**	10.75 ± 3.25
C63	Aggregative, Strong	59 (80)	21 (80)	44 (80)	36 (80)	**64.5 ± 9.5**	35.5 ± 9.5
C66	Aggregative, Strong	51 (80)	29 (80)	65 (80)	15 (80)	**72.5 ± 8.5**	27.5 ± 8.5
C73	Aggregative, Strong	79 (80)	1 (80)	78 (80)	2 (80)	**98.13 ± 0.625**	1.876 ± 0.625
C74	Aggregative, Moderate	1 (80)	73 (80)	1 (80)	61 (80)	1.25 ± 0	**83.75 ± 7.5**
C76	Aggregative, Moderate	0 (80)	37 (80)	0 (80)	53 (80)	0 ± 0	**56 ± 10**
C77	Aggregative, Moderate	2 (80)	74 (80)	0 (80)	76 (80)	1.25 ± 1.25	**93.75 ± 1.25**
C78	Aggregative, Strong	45 (80)	29 (80)	41 (80)	38 (80)	**53.5 ± 2.5**	42 ± 6
C80	Aggregative, Moderate	9 (80)	71 (80)	16 (80)	63 (80)	15.5 ± 4.5	**84 ± 5**
C83	Aggregative, Moderate	17 (80)	63 (80)	7 (80)	73 (80)	15 ± 6	**85 ± 6**
C85	Aggregative, Moderate	3 (80)	77 (80)	0 (80)	80 (80)	1.875 ± 1.875	**98.13 ± 1.875**
C87	Aggregative, Moderate	12 (80)	68 (80)	13 (80)	67 (80)	15.5 ± 0.5	**84.5 ± 0.5**
C88	Aggregative, Moderate	2 (80)	73 (80)	2 (80)	67 (80)	2.5 ± 0	**87.5 ± 3.5**
C90	Aggregative, Strong	61 (80)	19 (80)	54 (80)	26 (80)	**71.75 ± 4.25**	28.25 ± 4.25

C93	Aggregative, Strong	79 (80)	1 (80)	76 (80)	4 (80)	**96.88 ± 1.875**	3.125 ± 1.875
C94	Aggregative, Moderate	44 (80)	36 (80)	33 (80)	47 (80)	48 ± 7	**52 ± 7**
C96	Aggregative, Moderate	2 (80)	78 (80)	0 (80)	80 (80)	1.25 ± 1.25	**98.75 ± 1.25**
C98	Aggregative, Strong	53 (80)	27 (80)	44 (80)	36 (80)	**60.5 ± 5.5**	39.5 ± 5.5
C99	Aggregative, Strong	49 (80)	31 (80)	57 (80)	23 (80)	**66 ± 5**	34 ± 5
C101	Aggregative, Strong	44 (80)	36 (80)	42 (80)	38 (80)	**54 ± 1**	47 ± 1.5
C104	Aggregative, Strong	46 (80)	34 (80)	48 (80)	32 (80)	**58.75 ± 1.25**	41.25 ± 1.25
Controls:							
JEONG-1266	Aggregative, Strong	65 (80)	11 (80)	76 (80)	4 (80)	**88 ± 7**	9.5± 4.5
Sakai	Diffuse, Moderate	5 (80)	66 (80)	2 (80)	70 (80)	4.5 ± 1.5	**85.5 ± 2.5**
EC4115	Aggregative, Strong	63 (80)	17 (80)	65 (80)	15 (80)	**80 ± 1**	20 ± 1
SS17	Aggregative, Strong	67 (80)	13 (80)	77 (80)	3 (80)	**90 ± 6**	10 ± 6
SS 52	Aggregative, Strong	80 (80)	0	80 (80)	0	**100 ± 6**	0
EDL933	Aggregative, Moderate	22 (80)	58 (80)	25 (80)	55 (80)	29.5 ± 1.5	**70.5 ± 1.5**

[1] Each trial had one slide per bacterial group. Each slide in turn had 4 technical replicates spotted on it; 20 well-dispersed cells were evaluated per spot.

[2] MOI, multiplicity of infection

[3] Number of bacteria adhering to each cell is shown as a range of >10, and 1-10. Number of cells without bacteria is not shown

[4] Total number of cells evaluated in each trial is shown in parenthesis

[5] Percent means for ranges used to determine "strong, moderate or non-adherent" quantitative adherence are shown in bold.

Table S6. Adherence patterns of SS-O157 (n=53) isolates and control O157 (n=6) strains on Hep-2 cells.

Strains	Bacterial Adherence Pattern	Eukaryotic cells with adherent bacteria, in the ranges shown, for two different trials[1] (MOI[2] = 10^6 bacteria:10^5 cells)				Percent Mean +/- standard error of mean, of eukaryotic cells with adherent bacteria in the ranges shown[5]	
		Trial I		Trial II			
		>10	1-10[(3)]	>10	1-10	>10	1-10
C1	Diffuse, Moderate	0 (80)	40 (80)	0 (80)	55 (80)	0	**59.5 ± 9.5**
C2	Non-Adherent	2 (80)	21 (80)	0 (80)	22 (80)	1.25± 1.25	27± 1
C4	Non-Adherent	0 (80)	35 (80)	0 (80)	32 (80)	0	42± 2
C5	Diffuse, Moderate	0 (80)	33 (80)	3 (80)	47 (80)	1.9 ± 1.9	**50 ± 9**
C6	Diffuse, Moderate	0 (80)	53 (80)	0 (80)	50 (80)	0	**65 ± 2**
C7	Diffuse, Moderate	0 (80)	38 (80)	1 (80)	46 (80)	0.6 ± 0.6	**53 ± 5**
C9	Diffuse, Moderate	0 (80)	39 (80)	0 (80)	42 (80)	0	**51 ± 2**
C11	Diffuse, Moderate	0 (80)	37 (80)	0 (80)	54 (80)	0	**57 ± 11**
C12	Diffuse, Moderate	0 (80)	39 (80)	0 (80)	51 (80)	0	**56.5 ± 7.5**
C13	Non-Adherent	0 (80)	42 (80)	0 (80)	53 (80)	2.5 ± 2.5	33.5 ± 0.5
C14	Diffuse, Moderate	7 (80)	38 (80)	3 (80)	43 (80)	6.4 ± 2.6	**51 ± 3**
C15	Diffuse, Moderate	0 (80)	43 (80)	0 (80)	55 (80)	0	**61.5 ± 7.5**
C16	Diffuse, Moderate	0 (80)	47 (80)	7 (80)	41 (80)	4.5 ± 4.5	**55 ± 4**
C17	Diffuse, Moderate	1 (80)	49 (80)	4 (80)	31 (80)	3 ± 2	**50 ± 11**
C18	Diffuse, Moderate	0 (80)	43 (80)	0 (80)	39 (80)	0	**51.5 ± 2.5**
C22	Diffuse, Moderate	0 (80)	43 (80)	0 (80)	37 (80)	0	**50 ± 4**
C23	Diffuse, Moderate	0 (80)	53 (80)	0 (80)	38 (80)	0	**57 ± 9**
C28	Diffuse, Moderate	0 (80)	41 (80)	0 (80)	44 (80)	0	**53 ± 2**
C31	Diffuse, Moderate	0 (80)	42 (80)	0 (80)	39 (80)	0	**51 ± 2**

C32	Diffuse, Moderate	0 (80)	41 (80)	0 (80)	38 (80)	0	**49.5 ± 1.5**
C34	Diffuse, Moderate	1 (80)	40 (80)	1 (80)	40 (80)	1.25 ± 0	**50 ± 0**
C35	Diffuse, Moderate	0 (80)	43 (80)	0 (80)	42 (80)	0	**53.5 ± 0.5**
C36	Diffuse, Moderate	0 (80)	43 (80)	2 (80)	56 (80)	1.25 ± 1.25	**62 ± 8**
C38	Diffuse, Moderate	0 (80)	34 (80)	0 (80)	44 (80)	0	**49 ± 6**
C39	Diffuse, Moderate	0 (80)	45 (80)	0 (80)	48 (80)	0	**58 ± 2**
C40	Diffuse, Moderate	0 (80)	47 (80)	0 (80)	49 (80)	0	**60± 1**
C42	Diffuse, Moderate	0 (80)	59 (80)	1 (80)	55 (80)	0.6 ± 0.6	**71.5 ± 2.5**
C45	Non-Adherent	0 (80)	30 (80)	0 (80)	35 (80)	0	41 ± 3
C49	Non-Adherent	0 (80)	29 (80)	0 (80)	36 (80)	0	41 ± 5
C53	Diffuse, Moderate	0 (80)	40 (80)	0 (80)	54 (80)	0	**59 ± 9**
C54	Diffuse, Moderate	0 (80)	31 (80)	0 (80)	49 (80)	0	**50 ± 11**
C57	Diffuse, Moderate	0 (80)	45 (80)	0 (80)	60 (80)	0	**65.5 ± 9.5**
C62	Diffuse, Moderate	0 (80)	36 (80)	0 (80)	44 (80)	0	**50 ± 5**
C63	Diffuse, Moderate	0 (80)	42 (80)	0 (80)	38 (80)	0	**50 ± 3**
C66	Diffuse, Moderate	0 (80)	37 (80)	0 (80)	43 (80)	0	**50 ± 4**
C73	Diffuse, Moderate	0 (80)	35 (80)	0 (80)	48 (80)	0	**52 ± 8**
C74	Diffuse, Moderate	0 (80)	35 (80)	0 (80)	45 (80)	0	**50 ± 6**
C76	Diffuse, Moderate	0 (80)	60 (80)	0 (80)	61 (80)	0	**75.5 ± 0.5**
C77	Non-Adherent	0 (80)	22 (80)	0 (80)	26 (80)	0	30.5 ± 2.5
C78	Diffuse, Moderate	0 (80)	31 (80)	0 (80)	49 (80)	0	**50 ± 11**
C80	Diffuse, Moderate	0 (80)	48 (80)	0 (80)	34 (80)	0	**51.5 ± 8.5**
C83	Diffuse, Moderate	0 (80)	40 (80)	2 (80)	40 (80)	1.25 ± 1.25	**50**
C85	Diffuse, Moderate	3 (80)	64 (80)	15 (80)	57 (80)	11.4 ± 7.6	**75.5 ± 4.5**
C87	Diffuse, Moderate	4 (80)	67 (80)	0 (80)	61 (80)	2.5 ± 2.5	**80 ± 4**
C88	Diffuse, Moderate	0 (80)	36 (80)	0 (80)	51 (80)	0	**54.5 ± 9.5**

C90	Diffuse, Moderate	0 (80)	45 (80)	0 (80)	44 (80)	0	**55.5 ± 0.5**
C93	Diffuse, Moderate	0 (80)	31 (80)	0 (80)	49 (80)	0	**50 ± 11**
C94	Diffuse, Moderate	0 (80)	40 (80)	4 (80)	50 (80)	2.5 ± 2.5	**56.5 ± 6.5**
C96	Diffuse, Moderate	0 (80)	34 (80)	0 (80)	46 (80)	0	**50.5 ± 7.5**
C98	Diffuse, Moderate	0 (80)	51 (80)	1 (80)	29 (80)	0.6 ± 0.6	**50 ± 14**
C99	Diffuse, Moderate	0 (80)	46 (80)	0 (80)	58 (80)	0	**65.5 ± 7.5**
C101	Diffuse, Moderate	0 (80)	40 (80)	0 (80)	46 (80)	0	**54 ± 4**
C104	Diffuse, Moderate	1 (80)	48 (80)	0 (80)	57 (80)	0.6 ± 0.6	**65.5 ± 5.5**
**Controls:**							
JEONG-1266	Diffuse, Moderate	0 (80)	43 (80)	0 (80)	40 (80)	0	**52 ± 2**
Sakai	Diffuse, Moderate	0 (80)	43 (80)	0 (80)	55 (80)	0	**61.5 ± 7.5**
EC4115	Diffuse, Moderate	0 (80)	47 (80)	0 (80)	44 (80)	0	**57 ± 2**
SS17	Diffuse, Moderate	10 (80)	69 (80)	8 (80)	60 (80)	11.5 ± 1.5	**84.5 ± 1.5**
SS 52	Diffuse, Moderate	0 (80)	50 (80)	0 (80)	71 (80)	0	**76 ± 13**
EDL933	Diffuse, Moderate	0 (80)	40 (80)	4 (80)	46 (80)	2.5 ± 2.5	**54 ± 4**

[1] Each trial had one slide per bacterial group. Each slide in turn had 2 chambers (technical replicate) on it; 20 well-dispersed cells in 4 different fields were evaluated per chamber.
[2] MOI, multiplicity of infection
[3] Number of bacteria adhering to each cell is shown as a range of >10, and 1-10. Number of cells without bacteria is not shown
[4]Total number of cells evaluated in each trial is shown in parenthesis
[5] Percent means for ranges used to determine "strong, moderate or non-adherent" quantitative adherence are shown in bold.

Table S7. AST profiles of 53 SS-O157 isolates against 17 antibiotics[a, b]

SS-O157	AmC-30	AM-10	AZM-15	CL-10	GM-10	NA-30	PB-300	S-10	XNL-30	CRO-30	C-30	G-.25	TE-30	FOX-30	CIP-5	FOS-200	SXT-25	AST profile
C-1; C15; C40	S	S	S	S	S	S	S	S	S	S	S	S	S	S	S	S	S	Susceptible to all
C-2; C-6; C-57; C-62; C-73; C-74	S	S	I	S	S	S	S	I	S	S	S	I	S	S	S	S	S	AZM; S; G
C-4	S	S	I	S	S	S	S	S	S	S	S	R	I	S	S	S	S	AZM; G; TE
C-5	S	S	I	S	S	S	S	S	S	S	I	I	S	S	S	S	S	AZM; C; G
C-7	S	S	S	S	S	S	S	S	S	S	S	S	I	S	S	S	S	TE
C-9	S	S	I	S	S	S	S	I	I	S	S	S	S	S	S	S	S	AZM; S; XNL
C-11	S	S	I	S	S	S	S	S	I	S	I	R	S	S	S	S	S	AZM; XNL; C; G
C-12	S	S	I	S	S	S	S	S	I	S	I	R	I	S	S	S	S	AZM; XNL; C; G; TE
C-13	S	S	I	S	S	S	S	S	S	S	S	I	S	S	S	S	S	AZM; G
C-14; C-16; C-17; C-93	S	S	I	S	S	S	S	I	S	S	I	I	S	S	S	S	S	AZM; S; C; G
C-18; C-23; C-28; C-31; C-34; C-36; C-39; C-42; C-53; C-54; C-76; C-78	S	S	I	S	S	S	S	I	S	S	S	R	S	S	S	S	S	AZM; S; G
C-19	S	S	I	S	S	S	S	I	I	S	I	S	S	S	S	S	S	AZM; S; XNL; C
C-22	S	S	I	S	I	S	S	I	S	S	S	R	S	S	S	S	S	AZM; GM; S; G;
C-32	S	S	I	S	S	S	S	I	S	S	I	S	S	S	S	S	S	AZM; S; C
C-35; C-77; C-83; C-94	S	S	I	S	S	S	S	I	S	S	S	S	S	S	S	S	S	AZM; S
C-38; C-101	S	S	I	S	S	S	S	I	I	S	I	R	I	S	S	S	S	AZM; S; XNL; C; G; TE
C-45	S	S	I	S	S	S	S	I	I	S	S	R	S	S	S	S	S	AZM; S; XNL; G
C-49	S	S	I	S	S	S	S	I	S	S	I	R	S	S	S	S	S	AZM; S; C; G
C-66	S	S	I	S	S	I	S	S	I	S	I	R	S	S	S	S	S	AZM; NA; XNL; C; G
C-80	S	S	S	S	S	S	S	S	S	S	S	R	S	S	S	S	S	G
C-85	S	S	I	S	S	S	S	S	I	S	S	R	S	S	S	S	S	AZM; XNL; G
C-87	S	S	I	S	S	S	S	I	I	S	S	R	S	S	S	S	S	AZM; S; XNL; G
C-88	S	I	I	S	S	S	S	I	I	S	S	R	S	S	S	S	S	AM; AZM; S; XNL; G
C-90; C-98	S	S	I	S	S	S	S	S	S	S	S	R	S	S	S	S	S	AZM; G
C-96	S	S	I	S	S	S	S	S	S	S	I	R	S	S	S	S	S	AZM; C; G
C-99	S	S	I	S	S	S	S	S	I	S	S	R	S	S	S	S	S	AZM; XNL; G
C-104	S	S	S	S	S	S	S	I	S	S	S	R	R	S	S	S	S	S; G; TE

[a] Antibiotic Disc-Concentration (mcg); AmC-30 = Amoxicillin with Clavulanic Acid, 20/10 mcg; AM-10 = Ampicillin; AZM-15 =Azithromycin; FOX-30 = Cefoxitin; XNL-30 = Ceftiofur; CRO-30 = Ceftriaxone; CIP-5 = Ciprofloxacin; CL-10 = Colistin; C-30 = Chloramphenicol; FOS200 = Fosfomycin; GM-10 = Gentamicin; NA-30 = Nalidixic Acid; PB-300 = Polymyxin B; S-10 = Streptomycin; SXT = Sulfamethoxazole with Trimethoprim, 23.75/1.25 mcg; G-.25 = Sulfisoxazole; TE-30 = Tetracycline

[b] S: Susceptible, I: Intermediate susceptibility, R: Resistant

Table S8. AST profiles of six control O157 strains against 17 antibiotics [a, b]

Control O157 Strains	AmC-30	AM-10	AZM-15	CL-10	GM-10	NA-30	PB-300	S-10	XNL-30	CRO-30	C-30	G-.25	TE-30	FOX-30	CIP-5	FOS-200	SXT-25	AST profile
JEONG-1266	S	S	I	S	S	S	S	I	S	S	I	I	S	S	S	S	S	AZM; S; C; G;
Sakai	S	S	R	S	S	I	S	S	S	S	I	R	S	S	S	S	S	AZM; NA; C; G;
EC4115	S	S	I	S	S	S	S	S	S	S	I	S	I	I	S	S	S	AZM; C;TE; FOX
SS17	S	S	R	S	S	S	S	I	S	S	I	S	I	S	S	S	S	AZM; S; C; TE
SS 52	S	S	I	S	S	S	S	I	S	S	S	I	I	I	S	S	S	AZM; S; G; TE; FOX
EDL933	S	S	R	S	S	S	S	S	S	S	I	I	I	I	S	S	S	AZM; C; G; TE; FOX

[a] Antibiotic Disc-Concentration (mcg); AmC-30 = Amoxicillin with Clavulanic Acid, 20/10 mcg; AM-10 = Ampicillin; AZM-15 = Azithromycin; FOX-30 = Cefoxitin; XNL-30 = Ceftiofur; CRO-30 = Ceftriaxone; CIP-5 = Ciprofloxacin; CL-10 = Colistin; C-30 = Chloramphenicol; FOS200 = Fosfomycin; GM-10 = Gentamicin; NA-30 = Nalidixic Acid; PB-300 = Polymyxin B; S-10 = Streptomycin; SXT = Sulfamethoxazole with Trimethoprim, 23.75/1.25 mcg; G-.25 = Sulfisoxazole; TE-30 = Tetracycline

[b] S: Susceptible, I: Intermediate susceptibility, R: Resistant

Table S9. AST profiles of 20 bovine *E. coli* isolates against 17 antibiotics [a, b]

E. coli isolates	AmC-30	AM-10	AZM-15	CL-10	GM-10	NA-30	PB-300	S-10	XNL-30	CRO-30	C-30	G-.25	TE-30	FOX-30	CIP-5	FOS-200	SXT-25	AST profile
870-1; 887-1; 887-2; 887-3	S	S	I	S	S	S	S	I	S	S	S	S	I	S	S	S	S	AZM; S; TE
870-2; 870-3	I	I	I	S	S	S	S	R	S	S	R	R	R	S	S	S	S	AMC; AM; AZM; S; C; G; TE
870-4	S	S	I	S	R	S	S	I	S	S	S	S	I	S	S	S	S	AZM; GM; S; TE
887-4	S	S	I	S	S	S	S	I	S	S	R	S	I	S	S	S	S	AZM; S; C; TE
888-1	S	S	I	S	S	S	S	S	S	S	S	S	R	S	S	S	S	AZM; G
888-2	I	S	S	S	S	S	S	I	S	S	S	R	S	S	S	S	S	AMC; S; G
888-3	S	S	I	S	S	S	S	S	S	S	S	S	I	S	S	S	S	AZM; TE
888-4	S	S	I	S	S	S	S	I	S	S	S	R	S	S	S	S	S	AZM; S; G
912-1	S	S	I	S	S	I	S	I	S	S	S	R	S	S	S	S	S	AZM; NA; S; G
912-2; 912-3	S	S	I	S	S	S	S	I	S	S	S	R	R	S	S	S	S	AZM; S; G; TE
912-4	S	S	R	S	S	I	S	R	S	S	S	R	R	S	S	S	S	AZM; NA; S; G; TE
914-1	S	S	I	S	S	S	S	I	S	S	S	S	I	I	S	S	S	AZM; S; TE; FOX
914-2	S	I	I	S	S	S	S	I	S	S	S	S	S	S	S	S	S	AM; AZM; S
914-3	I	I	I	S	S	S	S	I	S	S	S	I	S	S	S	S	S	AMC; AM; AZM; S; G
914-4	I	I	I	S	S	S	S	I	S	S	S	S	S	S	S	S	S	AMC; AM; AZM; S

[a] Antibiotic Disc-Concentration (mcg); AmC-30 = Amoxicillin with Clavulanic Acid, 20/10 mcg; AM-10 = Ampicillin; AZM-15 = Azithromycin; FOX-30 = Cefoxitin; XNL-30 = Ceftiofur; CRO-30 = Ceftriaxone; CIP-5 = Ciprofloxacin; CL-10 = Colistin; C-30 = Chloramphenicol; FOS200 = Fosfomycin; GM-10 = Gentamicin; NA-30 = Nalidixic Acid; PB-300 = Polymyxin B; S-10 = Streptomycin; SXT = Sulfamethoxazole with Trimethoprim, 23.75/1.25 mcg; G-.25 = Sulfisoxazole; TE-30 = Tetracycline

[b] S: Susceptible, I: Intermediate susceptibility, R: Resistant

Table S10. AR and integrase gene profiles of SS-O157 (n=53) isolates [a, b]

SS-O157	Antibiotic resistance and toxin genes tested among SS O157 strains																																
	aac (3)-IV	*aad A1*	*acrB*	*ais*	*arnA*	*catA1*	bla_{CTX-M}	*dfrA1*	*dhfrI*	*emrA*	*fsr*	*int*	*macA*	*marA*	*mcr1*	*mcr2*	*mdtH*	*mdtO*	*mph(A)*	*pmrD*	*qnrA*	*rarD*	*stx1*	*stx2*	*sul1*	*sul2*	*sul3*	bla_{TEM}	*tetA*	*tetB*	*tetC*	*yfbH*	*yjcP*	*yjcR*
C-1	0	0	1	1	1	0	0	0	0	1	1	1	1	0	0	0	1	0	0	1	0	1	0	1	0	0	0	0	0	0	0	1	1	1
C-2	0	0	1	1	1	0	0	0	0	1	1	1	1	1	0	0	1	1	0	1	0	1	0	1	0	0	0	0	0	0	0	1	1	1
C-4	0	0	1	1	1	0	0	0	0	1	1	1	1	1	0	0	1	1	0	1	0	1	1	1	0	0	0	0	0	0	0	1	1	1
C-5	0	0	1	1	1	0	0	0	0	1	1	1	1	1	0	0	1	1	0	1	0	1	0	1	0	1	0	0	0	0	0	1	1	1
C-6	0	0	1	1	1	0	0	0	0	1	1	1	1	1	0	0	1	1	0	1	0	1	0	1	0	0	0	0	0	0	0	1	1	1
C-7	1	0	1	1	1	0	0	0	0	1	1	1	1	1	0	0	1	1	0	1	0	1	0	1	0	0	0	0	0	0	0	1	1	1
C-9	0	0	1	1	1	0	0	0	0	1	1	1	1	1	0	0	1	1	0	1	0	1	1	0	0	0	0	0	0	0	0	1	1	1
C-11	0	0	1	1	1	0	0	0	0	1	1	1	1	1	0	0	1	1	0	1	0	1	1	1	0	0	0	0	0	0	0	1	1	1
C-12	0	0	1	1	1	0	0	0	0	1	1	1	1	1	0	0	1	1	0	1	0	1	1	1	0	0	0	0	0	0	0	1	1	1
C-13	0	0	1	1	1	0	0	0	0	1	1	1	1	1	0	0	1	1	0	1	0	1	1	1	0	0	0	0	0	0	0	1	1	1
C-14	0	0	1	1	1	0	0	0	0	0	0	0	1	1	0	0	1	1	0	0	0	0	0	1	0	0	0	0	0	0	0	1	1	1
C-15	0	0	1	1	1	0	0	0	0	1	1	1	1	1	0	0	1	1	0	1	0	1	0	1	0	0	0	0	0	0	0	1	1	1
C-16	0	0	1	1	1	0	0	0	0	0	0	0	1	1	0	0	1	1	0	0	0	0	0	1	0	0	0	0	0	0	0	1	1	1
C-17	0	0	1	1	1	0	0	0	0	1	1	1	1	1	0	0	1	1	0	1	0	1	1	1	0	0	0	0	0	0	0	1	1	1
C-18	0	0	1	1	1	0	0	0	0	1	1	1	1	1	0	0	1	1	0	1	0	1	1	1	0	0	0	0	0	0	0	1	1	1
C-19	0	0	1	1	1	0	0	0	0	1	1	1	1	1	0	0	1	1	0	1	0	1	1	1	0	0	0	0	0	0	0	1	1	1
C-22	0	0	1	1	1	0	0	0	0	1	1	1	1	1	0	0	1	1	0	1	0	1	0	1	0	0	0	0	0	0	0	1	1	1
C-23	0	0	1	1	1	0	0	0	0	0	0	0	1	1	0	0	1	1	0	1	0	1	1	1	0	0	0	0	0	0	0	1	1	1
C-28	0	0	1	1	1	0	0	0	0	1	1	1	1	1	0	0	1	1	0	1	0	1	0	1	0	0	0	0	0	0	0	1	1	1
C-31	0	0	1	1	1	0	0	0	0	1	1	1	1	1	0	0	1	1	0	1	0	1	0	1	0	0	0	0	0	0	0	1	1	1
C-32	0	0	1	1	1	0	0	0	0	1	1	1	1	1	0	0	1	1	0	1	0	1	1	1	0	0	0	0	0	0	0	1	1	1
C-34	0	0	1	1	1	0	0	0	0	1	1	1	1	1	0	0	1	1	0	1	0	1	0	1	0	0	0	0	0	0	0	1	1	1
C-35	0	0	1	1	1	0	0	0	0	1	1	1	1	1	0	0	1	1	0	1	0	1	1	1	0	0	0	0	0	0	0	1	1	1
C-36	0	0	1	1	1	0	0	0	0	1	1	1	1	1	0	0	1	1	0	1	0	1	1	1	0	0	0	0	0	0	0	1	1	1
C-38	0	0	1	1	1	0	0	0	0	1	1	1	1	1	0	0	1	1	0	1	0	1	0	1	0	0	0	0	0	0	0	1	1	1

C-39	0	0	1	1	1	0	0	0	0	1	1	1	1	1	0	0	1	1	0	1	0	1	0	1	0	0	0	0	0	0	0	1	1	1
C-40	0	0	1	1	1	0	0	0	0	1	1	1	1	1	0	0	1	1	0	1	0	1	1	1	0	0	0	0	0	0	0	1	1	1
C-42	0	0	1	1	1	0	0	0	0	1	1	1	1	1	0	0	1	1	0	1	0	1	0	1	0	0	0	0	0	0	0	1	1	1
C-45	0	0	1	1	1	0	0	0	0	1	1	1	1	1	0	0	1	0	0	1	0	1	0	1	0	0	0	0	0	0	0	1	1	1
C-49	0	0	1	1	1	0	0	0	0	0	0	0	1	1	0	0	1	1	0	1	0	1	0	1	0	0	0	0	0	0	0	1	1	1
C-53	0	0	1	1	1	0	0	0	0	1	1	1	1	1	0	0	1	1	0	1	0	1	0	1	0	0	0	0	0	0	0	1	1	1
C-54	0	0	1	1	1	0	0	0	0	1	1	1	1	1	0	0	1	1	0	1	0	1	0	1	0	0	0	0	0	0	0	1	1	1
C-57	0	0	1	1	1	0	0	0	0	1	1	1	1	1	0	0	1	1	0	1	0	1	1	1	0	0	0	0	0	0	0	1	1	1
C-62	0	0	1	1	1	0	0	0	0	1	1	1	1	1	0	0	1	1	0	1	0	1	0	1	0	0	0	0	0	0	0	1	1	1
C-66	0	0	1	1	1	0	0	0	0	1	1	1	1	1	0	0	1	1	0	1	0	1	1	1	0	0	0	0	0	0	0	1	1	1
C-73	0	0	1	1	1	0	0	0	0	1	1	1	1	1	0	0	1	1	0	1	0	1	0	1	0	0	0	0	0	0	0	1	1	1
C-74	0	0	1	1	1	0	0	0	0	1	1	1	1	1	0	0	1	1	0	1	0	1	0	1	0	0	0	0	0	0	0	1	1	1
C-76	0	0	1	1	1	0	0	0	0	1	1	1	1	1	0	0	1	1	0	1	0	1	0	1	0	0	0	0	0	0	0	1	1	1
C-77	0	0	1	1	1	0	0	0	0	1	1	1	1	1	0	0	1	1	0	1	0	1	0	1	0	0	0	0	0	0	0	1	1	1
C-78	0	0	1	1	1	0	0	0	0	1	1	1	1	1	0	0	1	1	0	1	0	1	0	1	0	0	0	0	0	0	0	1	1	1
C-80	0	0	1	1	1	0	0	0	0	1	1	1	1	1	0	0	1	1	0	1	0	1	1	1	0	0	0	0	0	0	0	1	1	1
C-83	0	0	1	1	1	0	0	0	0	1	1	1	1	1	0	0	1	1	0	1	0	1	1	1	0	0	0	0	0	0	0	1	1	1
C-85	0	0	1	1	1	0	0	0	0	1	1	1	1	1	0	0	1	1	0	1	0	1	0	1	0	0	0	0	0	0	0	1	1	1
C-87	0	0	1	1	1	0	0	0	0	1	1	1	1	1	0	0	1	1	0	1	0	1	1	1	0	0	0	0	0	0	0	1	1	1
C-88	0	0	1	1	1	0	0	0	0	1	1	1	1	1	0	0	1	1	0	1	0	1	0	1	0	0	0	0	0	0	0	1	1	1
C-90	1	0	1	1	1	0	0	0	0	1	1	1	1	1	0	0	1	1	0	1	0	1	1	1	0	0	0	0	0	0	0	1	1	1
C-93	0	0	1	1	1	0	0	0	0	1	1	1	1	1	0	0	1	1	0	1	0	1	1	1	0	0	0	0	0	0	0	1	1	1
C-94	0	0	1	1	1	0	0	0	0	1	1	1	1	1	0	0	1	1	0	1	0	1	1	1	0	0	0	0	0	0	0	1	1	1
C-96	0	0	1	1	1	0	0	0	0	1	1	1	1	1	0	0	1	1	0	1	0	1	1	1	0	0	0	0	0	0	0	1	1	1
C-98	0	0	1	1	1	0	0	0	0	1	1	1	1	1	0	0	1	1	0	1	0	1	1	1	0	0	0	0	0	0	0	1	1	1
C-99	0	0	1	1	1	0	0	0	0	1	1	0	1	1	0	0	1	1	0	1	0	1	1	1	0	1	0	0	0	0	0	1	1	1
C-101	0	0	1	1	1	0	0	0	0	1	1	1	1	1	0	0	1	1	0	1	0	1	1	1	0	0	0	0	0	0	0	1	1	1
C-104	0	0	1	1	1	0	0	0	0	1	1	1	1	1	0	0	1	1	0	1	0	1	1	1	0	1	0	0	0	1	0	1	1	1

[a] 0, no amplicon; 1, amplicon present (See Table S7 for details on genes amplified)

[b] All isolates were Positive for following AR genes: *acrB, ais, arnA, macA, mdtH, yfbH, yjcP, yjcR*.

All isolates were Negative for following AR genes: *aadA1, catA1, bla_{CTXM}, dfrA1, dhfr1, mcr1, mcr2, mph(A), qnrA, sul1, sul3, bla_{TEM}, tetA, tetC*

Table S11. AR and integrase gene profiles of control O157 (n=6) strains [a, b]

Control O157	Antibiotic resistance and virulence genes tested among control O157 strains																																
	aac(3)-IV	*aadA1*	*acrB*	*ais*	*arnA*	*catA1*	*bla*$_{CTXM}$	*dfrA1*	*dhfrI*	*emrA*	*fsr*	*Int*	*macA*	*marA*	*mcr1*	*mcr2*	*mdtH*	*mdtO*	*mph(A)*	*pmrD*	*qnrA*	*rarD*	*stx*1	*stx*2	*sul1*	*sul2*	*sul3*	*bla*$_{TEM}$	*tetA*	*tetB*	*tetC*	*yfbH*	*yjcP*	*yjcR*
JEONG-1266	0	0	1	1	1	0	0	0	0	1	1	1	1	1	0	0	1	1	0	1	0	1	0	1	0	1	0	0	0	0	0	1	1	1
Sakai	0	0	1	1	1	0	0	0	0	1	1	1	1	1	0	0	1	1	0	1	0	1	1	1	0	1	0	0	0	0	0	1	1	1
EC4115	0	0	1	1	1	0	0	0	0	1	1	1	1	1	0	0	1	1	0	1	0	1	0	1	0	0	0	0	0	0	0	1	1	1
SS 17	0	0	1	1	1	0	0	0	0	1	1	1	1	1	0	0	1	1	0	1	0	1	0	1	0	0	0	0	0	0	0	1	1	1
SS 52	0	0	1	1	1	0	0	0	0	1	1	1	1	1	0	0	1	1	0	1	0	1	0	1	0	1	0	0	0	0	0	1	1	1
EDL 933	0	0	1	1	1	0	0	0	0	1	1	1	1	1	0	0	1	1	0	1	0	1	1	1	0	1	0	0	0	0	0	1	1	1

[a] 0, no amplicon; 1, amplicon present (See Table S7 for details on genes amplified)

[b] All isolates were Positive for following AR genes: *acrB, ais, arnA, emrA, fsr, Int, macA, marA, mdtH, mdtO, pmrD, rarD, stx*2*, yfbH, yjcP, yjcR*

All isolates were Negative for following AR genes: *aac(3)-IV, aadA1, catA1, bla*$_{CTXM}$*, dfrA1, dhfrI, mcr1, mcr2, mph(A), qnrA, sul1, sul3, bla*$_{TEM}$*, tetA, tetB, tetC*

Table S12. AR and integrase gene profiles of 20 bovine *E. coli* isolates[a, b]

	Antibiotic resistance and virulence genes tested among generic *E. coli* isolates																																
Bovine E. coli	*aac (3)-IV*	*aadA1*	*acrB*	*ais*	*arnA*	*catA1*	*bla*$_{CTXM}$	*dfrA*	*dhfrI*	*emrA*	*fsr*	*Int*	*macA*	*marA*	*mcr1*	*mcr2*	*mdtH*	*mdtO*	*mph(A)*	*pmrD*	*qnrA*	*rarD*	*stx1*	*stx2*	*sul1*	*sul2*	*sul3*	*bla*$_{TEM}$	*tetA*	*tetB*	*tetC*	*yfbH*	*yjcP*	*yjcR*
870-1	0	0	1	1	1	0	0	0	0	1	1	0	1	1	0	0	1	1	0	1	0	1	0	0	0	0	0	0	0	0	1	1	1	1
870-2	0	0	1	1	1	0	0	0	0	1	1	0	1	1	0	0	1	1	0	1	0	1	0	0	0	1	0	0	1	0	0	1	1	1
870-3	0	0	1	1	1	0	0	0	0	1	1	0	1	1	0	0	1	1	0	1	0	1	0	0	0	1	0	0	1	0	0	1	1	1
870-4	0	0	1	1	1	0	0	0	0	1	1	0	1	1	0	0	1	1	0	1	0	1	0	0	0	0	0	0	1	0	1	1	1	1
887-1	0	0	1	1	1	0	0	0	0	1	1	1	1	1	0	0	1	1	0	1	0	1	0	1	0	0	0	0	0	0	0	1	1	1
887-2	0	0	1	1	1	0	0	0	0	1	1	1	1	1	0	0	1	1	0	1	0	1	0	1	0	0	0	0	0	0	0	1	1	1
887-3	0	0	1	1	1	0	0	0	0	1	1	1	1	1	0	0	1	1	0	1	0	1	0	1	0	0	0	0	0	0	0	1	1	1
887-4	0	0	1	1	1	0	0	0	0	1	1	1	1	1	0	0	1	1	0	1	0	1	0	1	0	0	0	0	0	0	0	1	1	1
888-1	0	0	1	1	1	0	0	0	0	1	1	0	1	0	0	0	1	1	0	1	0	1	0	0	0	0	0	0	1	0	1	1	1	1
888-2	0	0	1	1	1	0	0	0	0	1	1	1	1	1	0	0	1	1	0	1	0	1	0	0	0	0	0	0	0	0	0	1	1	1
888-3	0	0	1	1	1	0	0	0	0	1	1	0	1	1	0	0	1	1	0	1	0	1	0	0	0	0	0	0	1	0	1	1	1	1
888-4	0	0	1	1	1	0	0	0	0	1	1	0	1	1	0	0	1	1	0	1	0	1	0	0	0	0	0	0	0	0	0	1	1	1
912-1	0	1	1	1	1	0	0	0	0	1	1	1	1	1	0	0	1	1	0	0	0	1	0	0	0	0	0	0	1	1	1	1	1	1
912-2	0	1	1	1	1	0	0	0	0	1	1	1	1	1	0	0	1	1	0	0	0	1	0	0	0	0	0	0	0	1	0	1	1	1
912-3	0	0	1	1	1	0	0	0	0	1	1	0	1	1	0	0	1	1	0	1	0	1	0	0	0	0	0	0	1	0	0	1	1	1
912-4	0	1	1	1	1	0	0	0	0	1	1	1	1	1	0	0	1	1	0	0	0	1	0	0	0	0	0	0	0	1	0	1	1	1
914-1	0	0	1	1	1	0	0	0	0	1	1	1	1	1	0	0	1	1	0	1	0	1	0	0	0	0	0	0	0	1	0	1	1	1
914-2	0	0	1	1	1	0	0	0	0	1	1	1	1	1	0	0	1	1	0	1	0	1	0	0	0	0	0	0	1	0	0	1	1	1
914-3	0	0	1	1	1	0	0	0	0	1	1	1	1	1	0	0	1	1	0	1	0	1	0	0	0	0	0	0	0	0	0	1	1	1
914-4	0	0	1	1	1	0	0	0	0	1	1	1	1	1	0	0	1	1	0	1	0	1	0	0	0	0	0	0	0	0	0	1	1	1

[a] 0, no amplicon; 1, amplicon present (See Table S7 for details on genes amplified)

[b] All isolates were Positive for following AR genes: *acrB, ais, arnA, emrA, fsr, macA, mdtH, mdtO, rarD, yfbH, yjcP, yjcR*

All isolates were Negative for following AR genes: *aac(3)-IV, catA1, bla*$_{CTXM}$, *dfrA1, dhfrI, mcr1, mcr2, mph(A), qnrA, sul1, sul3, bla*$_{TEM}$

Table S13: AR and integrase gene sequencing results for representative SS-O157 isolates.

SS-O157	Gene	Gene Size (bp)	Sequencing (BLASTn) results
C42	*acrB*	838	aminoglycoside/multidrug transporter permease
C45	*acrB*	838	aminoglycoside/multidrug transporter permease
C45	*ais*	362	histidine phosphatase family protein
C45	*arnA*	241	bifunctional UDP-glucuronic acid oxidase/UDP-4-amino-4-deoxy-L-arabinose formyltransferase
C39	*dfrA1*	367	DUF1338 domain-containing protein
C45	*dfrA1*	367	DUF1338 domain-containing protein
C39	*emrA*	901	multidrug export protein EmrA
C90	*Fsr*	141	MFS transporter
C39	*Int*	545	integrase
C39	*macA*	791	macrolide transporter subunit MacA
C42	*marA*	128	MDR efflux pump AcrAB transcriptional activator MarA
C77	*mcr1*	780	ribonucleoside hydrolase RihC
C78	*mcr1*	780	ribonucleoside hydrolase RihC
C93	*mcr1*	780	ribonucleoside hydrolase RihC
C39	*mdtH*	392	MFS transporter
C90	*mdtO*	328	multidrug resistance protein MdtO
C90	*pmrD*	175	signal transduction protein PmrD
C90	*rarD*	370	protein RarD
C2; C45	*sul1*	479	autotransporter; adhesin
C104	*sul2*	348	sulfonamide-resistant dihydropteroate synthase Sul2
C2	*sul3*	264	C4-dicarboxylate transporter DcuC, ClpXP protease specificity-enhancing factor
C4	*tetA*	372	chromosomal replication initiation protein DnaA
C7	*tetA*	372	chromosomal replication initiation protein DnaA
C12	*tetA*	372	chromosomal replication initiation protein DnaA
C38	*tetA*	372	chromosomal replication initiation protein DnaA
C90	*tetA*	372	chromosomal replication initiation protein DnaA
C101	*tetA*	372	chromosomal replication initiation protein DnaA
C104	*tetA*	372	chromosomal replication initiation protein DnaA
C104	*tetB*	222	tetracycline efflux MFS transporter Tet(B)
C39	*yfbH*	543	4-deoxy-4-formamido-L-arabinose-phosphoundecaprenol deformylase
C45	*yjcP*	540	multidrug resistance outer membrane protein MdtP
C42	*yjcR*	621	multidrug transporter subunit MdtN

Table S14: AR gene sequencing results for representative bovine *E. coli* isolates and control O157 strains.

E. coli isolates	Gene	Gene Size (bp)	Sequencing (BLASTn) results
912-1	*aadA1*	447	ANT(3")-Ia family aminoglycoside nucleotidyl transferase AadA1
912-2	*aadA1*	447	aminoglycoside-3"-adenylyltransferase (aadA1)
887-1	*Ais*	362	histidine phosphatase family protein, Polymyxin resistance protein PmrG
888-1	*Ais*	362	aluminum inducible protein Ais
870-1	*arnA*	241	bifunctional polymyxin resistance arnA protein
912-2	*arnA*	241	bifunctional UDP-glucuronic acid oxidase/UDP-4-amino-4- deoxy-L-arabinose formyltransferase
887-3	*bla*$_{\mathrm{CTXM}}$	499	bifunctional chorismate mutase/prephenate dehydratase
887-2	*mcr1* (full length)	1626	type II secretion system protein, Gsp gene *E. coli*
887-3	*mcr1* (full length)	1626	allantoate amidohydrolase
Sakai	*mcr1* (full length)	1626	exonuclease subunit SbcC
EDL933	*mcr1* (full length)	1626	exonuclease subunit SbcC
EC4115	*mcr1* (full length)	1626	exonuclease subunit SbcC
JEONG-1266	*mcr1* (full length)	1626	exonuclease subunit SbcC
SS17	*mcr1* (full length)	1626	exonuclease subunit SbcC
SS 52	*mcr1* (full length)	1626	exonuclease subunit SbcC
Sakai	*sul2*	348	sulfonamide-resistant dihydropteroate synthase Sul2
870-2	*sul2*	348	sulfonamide-resistant dihydropteroate synthase Sul2
870-2	*tetA*	372	tetracycline efflux protein TetA
870-2	*tetA* (full length)	1103	tetracycline efflux protein TetA
912-3	*tetA*	372	tetracycline efflux protein TetA
912-1	*tetB*	222	tetracycline efflux MFS transporter Tet(B)
912-2	*tetB*	222	tetracycline efflux MFS transporter Tet(B)
870-4	*tetC*	379	tetracycline efflux MFS transporter Tet(C)
888-1	*tetC*	379	tetracycline repressor protein class C, TetC

Table S15. *Stx2* gene: Estimates of evolutionary divergence[a] over sequence pairs between two groups and within each group.

Comparison groups	Genetic Distance/Base substitution per site
Overall mean genetic distance	0.021
Mean diversity in entire population	0.022
Mean diversity within subpopulations	0.008
Mean inter population diversity	0.015
Between *E. coli* and control O157 groups	0.029
Between *E. coli* and SS O157 groups	0.038
Between control O157 and SS O157 groups	0.011
Within *E. coli* isolates group	0.012
Within control O157 isolates group	0.006
Within SS O157 isolates group	0.005

[a]Genetic distance (evolutionary divergence) and rate variation among sites analyses were conducted using the maximum likelihood statistical method and Jukes-Cantor nucleotide substitution model with a gamma distribution (shape parameter = 0.5)
