# Supplementary figures and images for: Supershed Escherichia coli O157:H7 Has Potential for Increased Persistence on the Rectoanal Junction Squamous Epithelial Cells and Antibiotic Resistance
Source: Int J Microbiol. 2020 Apr 13;2020:2368154. doi: 10.1155/2020/2368154 (PMC7178529; doi:10.1155/2020/2368154)

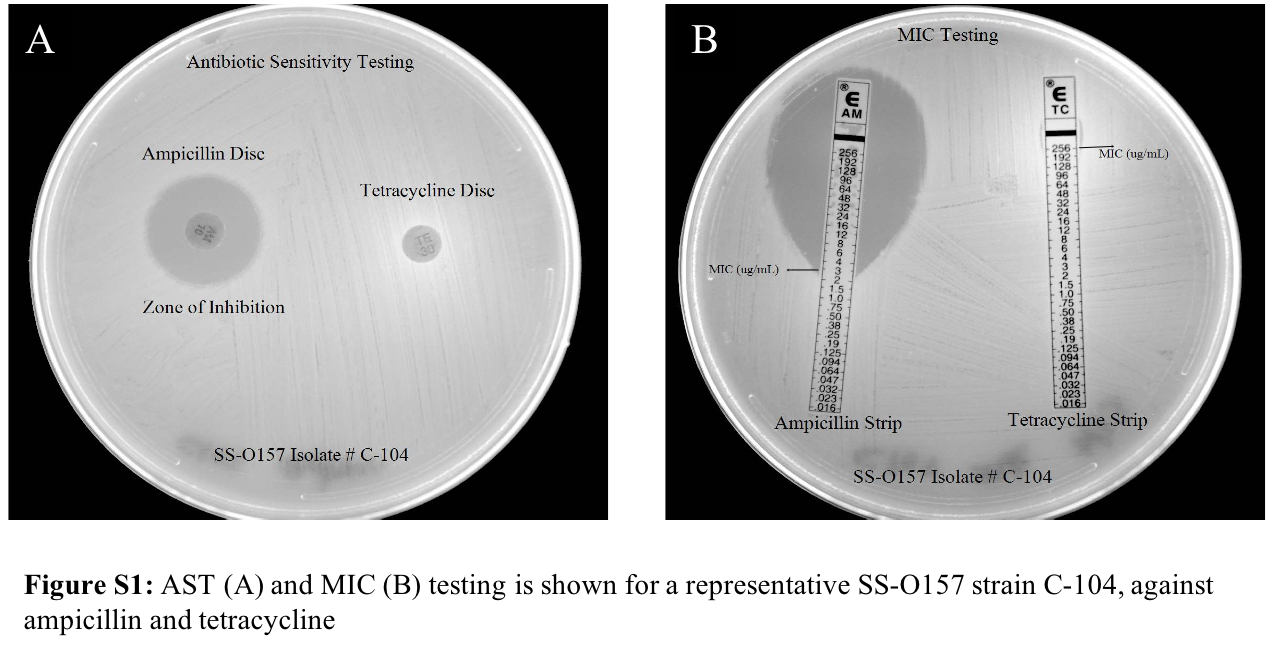

Supplement: Supplementary Materials — The supplementary files contain the supplementary tables supporting the results in the manuscript, and as listed in the same, include the following: Table S1: zone of inhibition diameters used to determine AST profiles of control Escherichia coli (ATCC®25922™) and all bacterial isolates tested in this assay. Table S2: CLSI break points for determining MIC (μg/mL) profiles of control Escherichia coli (ATCC®25922™) and all bacterial isolates tested in this assay. Table S3: primers used in this study. Table S4: PATS profiles of SS-O157 (n = 101) isolates. Table S5: adherence patterns of SS-O157 (n = 53) isolates and control O157 (n = 6) strains on RSE cells. Table S6: adherence patterns of SS-O157 (n = 53) isolates and control O157 (n = 6) strains on HEp-2 cells. Table S7: AST profiles of 53 SS-O157 isolates against 17 antibiotics. Table S8: AST profiles of six control O157 strains against 17 antibiotics. Table S9: AST profiles of 20 bovine E. coli isolates against 17 antibiotics. Table S10: AR and integrase gene profiles of SS-O157 (n = 53) isolates. Table S11: AR and integrase gene profiles of control O157 (n = 6) strains. Table S12: AR and integrase gene profiles of 20 bovine E. coli isolates. Table S13: AR and integrase gene sequencing results for representative SS-O157 isolates. Table S14: AR gene sequencing results for representative bovine E. coli isolates and control O157 strains. Table S15: Stx2 gene: estimates of evolutionary divergence over sequence pairs between two groups and within each group. Figure S1: AST (panel A) and MIC (panel B) testing is shown for a representative SS-O157 strain C-104 against ampicillin and tetracycline. Figure S2: nucleotide (A) and amino acid (B) sequence of the Stx2 genes amplified and sequenced from six control O157 and four bovine E. coli isolates. Sequences were aligned using ClustalW, multiple sequence alignment program. Figure S3: neighbor joining (A) and maximum likelihood (B) phylogenetic trees of Stx2 gene sequences fr [file 2368154.f1.zip › 2368154.f1/Fig S1_IJMICRO_3050785.tiff]
